# Disability weight measurement for the severity of different diseases in Wuhan, China

**DOI:** 10.1186/s12963-023-00304-y

**Published:** 2023-05-04

**Authors:** Xiaoxue Liu, Yan Guo, Fang Wang, Yong Yu, Yaqiong Yan, Haoyu Wen, Fang Shi, Yafeng Wang, Xuyan Wang, Hui Shen, Shiyang Li, Yanyun Gong, Sisi Ke, Wei Zhang, Qiman Jin, Gang Zhang, Yu Wu, Maigeng Zhou, Chuanhua Yu

**Affiliations:** 1 Department of Epidemiology and Biostatistics, School of Pubic Health, Wuhan University, 115 Donghu Road, Wuchang District, Wuhan, 430071 Hubei China; 2 Global Health Research Division, Public Health Research Center and Department of Public Health and Preventive Medicine, Wuxi School of Medicine, Jiangnan University, Wuxi, 214122 Jiangsu China; 3 Wuhan Centers for Disease Control and Prevention, Wuhan, 430024 Hubei China; 4 Department of Epidemiology and Biostatistics, School of Public Health, Xuzhou Medical University, Xuzhou, 221004 China; 5 School of Public Health and Management, Hubei University of Medicine, Shiyan, 442000 Hubei China; 6 National Center for Chronic and Non-communicable Disease Control and Prevention, Chinese Center for Disease Control and Prevention, Nanwei Road 27, Xicheng District, Beijing, 100050 China; 7 Global Health Institute, Wuhan University, Wuhan, 430072 Hubei China

**Keywords:** Disability weight, Disease burden, Sequela, Health state, Paired comparison

## Abstract

**Background:**

Measurement of the Chinese burden of disease with disability-adjusted life-years (DALYs) requires disability weight (DW) that quantify health losses for all non-fatal consequences of disease and injury. The Global Burden of Disease (GBD) 2013 DW study indicates that it is limited by lack of geographic variation in DW data and by the current measurement methodology. We aim to estimate DW for a set of health states from major diseases in the Wuhan population.

**Methods:**

We conducted the DW measurement study for 206 health states through a household survey with computer-assisted face-to-face interviews and a web-based survey. Based on GBD 2013 DW study, paired comparison (PC) and Population health equivalence (PHE) method was used and different PC/PHE questions were randomly assigned to each respondent. In statistical analysis, the PC data was analyzed by probit regression. The probit regression results will be anchored by results from the PHE data analyzed by interval regression on the DW scale units between 0 (no loss of health) and 1 (loss equivalent to death).

**Results:**

A total of 2610 and 3140 individuals were included in the household and web-based survey, respectively. The results from the total pooled data showed health state “mild anemia” (DW = 0.005, 95% UI 0.000–0.027) or “allergic rhinitis (hay fever)” (0.005, 95% UI 0.000–0.029) had the lowest DW and “heroin and other opioid dependence, severe” had the highest DW (0.699, 95% UI 0.579–0.827). A high correlation coefficient (Pearson’s* r* = 0.876; *P* < 0.001) for DWs of same health states was observed between Wuhan’s survey and GBD 2013 DW survey. Health states referred to mental symptom, fatigue, and the residual category of other physical symptoms were statistically significantly associated with a lower Wuhan’s DWs than the GBD’s DWs. Health states with disfigurement and substance use symptom had a higher DW in Wuhan population than the GBD 2013 study.

**Conclusions:**

This set of DWs could be used to calculate local diseases burden for health policy-decision in Wuhan population. The DW differences between the GBD’s survey and Wuhan’s survey suggest that there might be some contextual or culture factors influencing assessment on the severity of diseases.

**Supplementary Information:**

The online version contains supplementary material available at 10.1186/s12963-023-00304-y.

## Background

China is facing rapid rise of non-communicable diseases driven by urbanisation, rising incomes, and aging poses major challenges, as does a shift to chronic disability. Rapid transitions imposed on the health system by epidemiological and demographic change differed between Chinese provinces or regions [1, 2]. The population distribution of disease burden caused by risk factors exposure varies substantially in different provinces. Localized health policies need to be implemented to tackle the diverse challenges faced by local health-care systems. At present, it is very necessary to accurately calculate local disease burden. However, as reported, the task of achieving the Health China 2030 target would be daunting for two thirds of the provinces [3].

Disability weight (DW), a key basically parameter for calculating disease burden, is a weight factor that reflects the severity of health state from disease or injury. DW has a value between 0 (equivalent to full health) and 1 (equivalent to death). The estimation on DW has been continuously changed by modifying and adapting methodologies in previous studies [4–8]. Since 1990, a set of DWs constantly updated were used to estimate disability-adjusted life years (DALY) which iterated by Global Burden of Diseases (GBD) team yearly [4, 7, 9–13]. DALY is a summary measure of population health that captures health losses associated with mortality and with different non-fatal outcomes of diseases and injuries in a single figure. DALY is calculated by adding years of life lost (YLL) and years lived with disability (YLD) [13, 14]. To compute YLD for a particular health outcome in a population, the number of people living with that outcome is multiplied by cause-specific DW [6, 7, 9]. The cause-specific DW is also a basis for calculation of health-adjusted life expectancy (HALE) [15]. Until 2015, a set of 235 unique health states associated disease and injury were mapped to 0–1 of DW by GBD 2013 DW measurement study, using paired comparison (PC) and population health equivalence (PHE) approaches [6, 7]. However, the approaches used by the GBD 2013 study were also debated for the interpretation of evidence on the level of international agreement in PC responses [16–19]. Apart from that, previous studies suggested that DW valuation in East Asia regions might differ from that in Western countries [20]. The GBD 2013 study also pointed out that future country-specific survey data were needed to advance DW research as it was limited by lack of geographic variation in the data and by the current DW measurement methodology [7, 21]. Previous study reported that the DWs of specific stage of therapy, remission, metastasis, and terminal of all cancers in China were 0.310, 0.218, 0.450, and 0.653, respectively [22], which were relatively higher than the results in the GBD 2013 DW study [7].

For major diseases, reliable and comparable analyses of their disease burden are key measures to preventing disease and injury. Almost no study is carried out to accurately estimate disease burden in Wuhan city. Wuhan is the capital city of Hubei Province with very rapid economic development and aging population. The DW data for disease and injury were also very limited in Wuhan population. To support evidence-based policy development and targeted prevention and control of major diseases, the assessment on disease burden in Wuhan population is necessary and urgent. In this study, we aim to estimate DWs for a set of health states in Wuhan population, which could lay the foundation and provide evidence for health policy-decision on Hubei province and other regions of China.

## Methods

### Study design and participants

The study was conducted through household and web-based survey in Wuhan, China in the same way as the GBD 2013 DW study [6, 7]. The household survey was performed from November 1, 2019 to January 11, 2020, using computer-assisted face-to-face interviews. The web-based survey was conducted from May 12 to July 22, 2020 [23]. This study was approved by the Ethics Committee of Medical Department of Wuhan University (2019YF2055), and a waiver of written informed consent obtained from participants prior to survey participation was approved.

In our study, eligible participants were 18 years or older in household survey and 18–69 years of age in web-based survey. People aged over 70 were excluded in online survey because they were expected to be less familiar with the internet and find the survey too difficult. To confirm every possible pair of 206 health states evaluated with the PC questions, we considered that with 205 times 206 possible pairs of health states and 16 PC questions per respondent, the target sample sizes would result in at least 1 answer for comparison of each unique pair. In our study, the target number of study participants was set between around 2000 and 3000 in household survey. To consider a representative of Wuhan population, these respondents were drawn from the target population by using a multistage stratified random sampling method, with reference to age, sex, and socioeconomic status. There are 14 administrative districts and 1 functional district in Wuhan, China. According to the population and economic development, 15 districts were divided into three types: central districts (7), remote districts (4), and economic development zone (3). According to the proportion of population size, 3 streets (villages and towns) were randomly selected from the central and remote district separately, 1 or 2 streets (villages and towns) were randomly selected from the development zone, and a total of 38 community streets (villages and towns) were selected as investigation spots. Within each randomly selected street (villages and towns), 2 communities (village committees) were randomly selected. Within each community (village committee), 1–2 residents/village groups were randomly selected. In each group, all residents aged 18 and over in each household were surveyed.

For the web-based survey, we recruited participants through professional networks of the study investigators and staff from Wuhan Center for Disease Control and Prevention (CDC) [23]. We also announced the web-based survey on relevant websites, and allowed participants to recruit others via word of mouth. Each community residents received a link to the questionnaire via a personal WeChat message. Respondents were recruited in the web-based survey and randomly given US$ 0.3–15. In order to improve data quality, a series of measures were set up for quality control: (1) allowing a user to answer once only; (2) requiring a minimum survey completion time of 3 min; (3) excluding answers to the 16 PC questions are all A or B, and all answers alternating A and B.

### Health states and lay description used in the DW questionnaire

DW reflects the severity of disability caused by a disease or injury to the patient’s health and social functions. We tested DWs for a total of 206 health states which reflected a diversity of health outcomes caused by disease or injuries. Each health state was described by brief lay descriptions in terms of the functional loss or symptoms. For example, lung cancer has a sequela “metastatic phase of lung cancer”, and its health state “cancer, metastatic”, and its lay description “has severe pain, extreme fatigue, weight loss and high anxiety”. In our study, 172 health states and their lay descriptions were included from the GBD 2017 study [24] which used GBD 2013’s DW for 3484 sequelae and YLD estimates of 354 diseases and injuries. Different sequelae from diseases correspond to same, similar, or different health states. Thus, we removed duplicate lay descriptions of health states and keep 172 descriptions which corresponding to 172 health states. 32 lay descriptions of health states were included from the European DW study [6]. We also simplified two lay descriptions of original GBD health states (moderate and severe hearing loss). All of health states and lay descriptions in English are presented in the appendix [see Additional file 1: Table 2].

The lay descriptions of health states were firstly translated from the GBD 2013 DW study into Chinese by Liu X., Wang F., Wen H., Shi F., and Wang Y. Yu C. and Zhou M. revised them. These persons are native speakers with a medical background. Subsequently back translation was verified independently by bilingual native speaker and rechecked by Liu X. and Yu C. These lay descriptions of health states have a word limit of 75 words or less. The brief lay descriptions are developed to mainly focus on the major functional consequences and symptoms associated with the health state using simple, non-clinical vocabulary. We then consulted disease experts and health professionals to ensure that these descriptions were appropriate and reflective of the common manifestations of the disabling sequela.

### Survey procedure

In face-to-face household survey, the questionnaire included questions regarding socio-demographic and geographic characteristics of respondents, and 16 PC questions. The first part included gender, age, educational level, and other socio-demographic factors. Thereafter, the participants were randomly assigned health states with answering 16 PC questions, which was based on a computer-generated random selection of health states pairs, following a randomization algorithm based on the minimum number of selections that the health state pairs had at that moment. In this study, we assigned the same pair of health states in the third, 10th, and 16th PC questions to allow assessment of test–retest reliability and internal consistency of PC responses. The web survey added 3 questions for population health equivalence, according to GBD 2010 and European DW study [4, 6].

### Valuation method

We used PC and PHE methods on basis of previous DW studies [7]. For PC method, participants were asked to select the healthier option between two health states which were randomly extracted from 206 health states. The PHE method is used to compare the relationship between death and non-fatal outcomes by collecting equivalent health information. It asks respondents to compare the health benefits of two hypothetical life-saving or health-improving programs and choose which health program they think produced the greater overall population health benefit. In PHE question, the first health program prevented 1000 people from getting an illness that causes rapid death; the second health program prevented 1500, 2000, 3000, 5000, or 10 000 (randomly selected for the second program in each question) people from getting an illness that is nonfatal but causes the lifelong health problems of the randomly selected health states. In this study, a subset of 28 health states were estimated for PHE methods (see Additional file 1: Table 3). The severity of 28 health states ranged from mild to severe, including mild, moderate, and severe health states.

### Statistical analysis

The PC data from included respondents was included in probit regression models. The pooled PC data consists of data from household survey and web-based survey. Probit regression model has been commonly used for PC data. The PC method presented two health states to the respondents simultaneously, and the respondents compared the severity of the two health states and made a choice of 0 or 1—i.e., a binary response variable *Y* in the probit regression model; *Y* = 1 represents that the first health state in a paired comparison is chosen as the healthier one, and *Y* = 0 represents that the second is chosen as the healthier one. *X* is indicator variables for each health state. We ran probit regression analysis on the choice responses in paired comparison data, with indicator variables for each health state that took the value 1 for the first state in a paired comparison, -1 for the second state in a paired comparison, and 0 otherwise. This modelling strategy was used to infer the distances between values attached to different health states based on the observed frequencies of responses to paired comparison questions. A binary response variable *Y* was modeled:

$$P\left( {Y = 1{|}X} \right) = \Phi \left( {X^{\prime}\beta } \right)$$

where $\Phi$ is the cumulative distribution function of the standard normal distribution; *X* is a vector of explanatory variables; and parameters $\beta$ represents probit regression coefficients which are estimated by maximum likelihood.

The probit regression yielded predicted probabilities that captured the relative differences in health levels across health states, which were consistent with the PC responses. The regression results were not a 0–1 DW scale. To anchor the results of probit regression on PC data, we performed interval regression analysis to obtain predicted probabilities from PHE data. To link the predicted probabilities between the PC and DW estimates derived from the PHE, linear regression was applied with the DW estimates from PHE as the dependent variables and the predicted probabilities from the PC as the independent variables. We obtained the predicted probabilities by using the coefficient estimates of each health state and regarded them as DW estimates. Lastly, Monte Carlo integration using normal random samples was used to estimate the mean of DW estimates, and a bootstrapping approach with 1000 replicate samples was used to estimate their 95% uncertainty intervals (UI). The specific model and detailed methods could be found in GBD 2010 DW study [4].

We compared the set DWs of same health states between this study and the GBD 2013 DW study to assess that what symptoms mentioned in the lay descriptions of health state were associated with the DW difference. Based on the recent Japanese DW study, eleven identified symptom categories referred to the lay descriptions of health states, including mobility, pain, mental symptoms, fatigue, disfigurement, sensory symptoms, infection/diarrhoea, substance use, activities of daily living (ADL), cognitive symptoms, and other physical symptoms [25]. The identified symptom categories for 206 health states were presented in appendix [see Additional file 1: Table 2]. A linear regression model was used to analyze outcomes of proportional differences between Wuhan’s and previous DWs of 206 health states. The difference values “*d* = (China DW—GBD 2013 DW)/GBD 2013 DW $\times \hspace{0.17em}$100*”* as the dependent variable *Y*, and the 11 symptom categories corresponding to 206 health states as the binary independent variable X*i,* where *i* represents 1–11. The regression coefficient corresponding to X_*i*_ is positive, which means that the DW value of the disease symptoms mentioned in the health states description is higher than that of the comparison group. On the contrary, the negative coefficient means that it is lower than that of the comparison group. All eleven symptom categories were simultaneously entered into the liner regression model. We performed all statistical analysis with R (version 4.0.2) and Stata/MP (version 15). The Stata code is available from the author upon request. *P* values less than 0.05 were regarded statistically significant in this study.

### Role of funding source

Funding was provided by the National Natural Science Foundation of China, the National Key Research and Development Program of China, the Wuhan Medical Key Research Program of Joint Fund of Hubei Health Committee, and the 2020 Wuhan Municipal Health Commission Project. The funder had no role in writing the manuscript or the decision to submit for publication.

## Results

### Respondents

A total of 5750 participants were included in our study. There were 2610 respondents in household survey and 3140 respondents in web-based survey. Table 1 shows the details of total participants’ socio-demographic information. When compared with the Wuhan’s population, those who participated in this survey tended to have female gender and be younger. The age group of 30–49 accounted for about 48% of the total survey participants.

**Table 1:** Socio-demographic information for the study participants

	Wuhan’s population (%) ^a^	n = 5750	*P*^c^
*Age (years)*			
18–29	13.4%	1222 (21.2%)	0.097
30–49	33.1%	2770 (48.2%)	
50–69	27.5%	1573 (27.4%)	
≥ 70	9.2%	185 (3.2%)	
*Gender*			
Men	50.8%	2156 (37.5%)	0.056
Women	49.2%	3594 (62.5%)	
*Education level*			
Elementary school graduate or below	NA	459 (8.0%)	
Middle school graduate		778 (13.5%)	
High school graduate or attending college		1214 (21.1%)	
College graduate or above		3299 (57.4%)	
*Occupation*			
Non-manual	NA	2593 (45.1%)	
Manual		1170 (20.3%)	
Others^b^		1987 (34.6%)	

^a^According to Wuhan Statistical Yearbook compiled by Wuhan Municipal Statistics Bureau: http://tjj.wuhan.gov.cn/tjfw/tjnj/202112/t20211220_1877108.shtml (2020 population)

^b^Other occupation including housewife and student, soldier, out of work, retired, and other laborers

^c^*P* values resulting from the chi-square test

*NA* Not available in China Statistical Yearbook

### Estimates of disability weight

Table 2 shows the estimated DWs for the 206 health states in Wuhan population. In the GBD 2013 DW study, 83.0% of the health states were located below a DW of 0.4. The frequency distribution of the DW from this study slightly differed according to each survey (Table 3). The proportion of health states below a DW of 0.4 was 88.8% in Wuhan survey.

**Table 2:** Disability weights with uncertainty intervals (UI) for 206 health states in Wuhan

	Health states	Disability Weight (95% UI)
	Infectious disease	
1	Acute episode, mild	0.012 (0.001–0.054)
2	Acute episode, moderate	0.087 (0.022–0.202)
3	Acute episode, severe	0.11 (0.034–0.228)
4	Post-acute consequences (fatigue, emotional lability, and insomnia)	0.065 (0.013–0.169)
	Diarrhoea	
5	Mild	0.057 (0.011–0.152)
6	Moderate	0.148 (0.059–0.266)
7	Severe	0.238 (0.126–0.352)
8	Epididymo-orchitis	0.085 (0.022–0.197)
9	Herpes zoster	0.022 (0.002–0.083)
10	HIV: symptomatic, pre-AIDS	0.15 (0.059–0.271)
11	HIV/AIDS: receiving antiretroviral (ARV) treatment	0.049 (0.008–0.138)
12	AIDS: not receiving antiretroviral (ARV) treatment	0.392 (0.272–0.5)
13	Intestinal nematode infections: symptomatic	0.059 (0.012–0.164)
14	Lymphatic filariasis: symptomatic	0.154 (0.059–0.272)
15	Ear pain	0.032 (0.004–0.109)
	Tuberculosis	
16	Not HIV infected	0.292 (0.177–0.404)
17	HIV infected	0.39 (0.272–0.496)
	Cancer	
18	Diagnosis and primary treatment	0.166 (0.07–0.29)
19	Metastatic	0.29 (0.168–0.394)
	Terminal phase	
20	With medication (for cancers and end-stage kidney or liver disease)	0.568 (0.462–0.69)
21	Without medication (for cancers and end-stage kidney or liver disease)	0.344 (0.223–0.445)
	Cardiovascular and circulatory disease	
	Acute myocardial infarction	
22	Days 1–2	0.303 (0.185–0.407)
23	Days 3–28	0.068 (0.015–0.172)
	Angina pectoris	
24	Mild	0.032 (0.004–0.108)
25	Moderate	0.051 (0.009–0.144)
26	Severe	0.154 (0.064–0.27)
27	Cardiac conduction disorders and cardiac dysrhythmias	0.174 (0.074–0.293)
28	Claudication	0.016 (0.001–0.067)
	Heart failure	
29	Mild	0.052 (0.009–0.148)
30	Moderate	0.069 (0.014–0.175)
31	Severe	0.146 (0.055–0.271)
	Stroke	
32	Long-term consequences, mild	0.028 (0.003–0.098)
33	Long-term consequences, moderate	0.042 (0.006–0.123)
34	Long-term consequences, moderate plus cognition problems	0.097 (0.028–0.213)
35	Long-term consequences, severe	0.272 (0.153–0.377)
36	Long-term consequences, severe plus cognition problems	0.288 (0.172–0.402)
	Diabetes and digestive and genitourinary disease	
37	Diabetic neuropathy	0.081 (0.019–0.189)
38	Chronic kidney disease (stage IV)	0.049 (0.009–0.144)
	End-stage renal disease	
39	With kidney transplantation	0.043 (0.007–0.128)
40	On dialysis	0.571 (0.459–0.694)
41	Decompensated cirrhosis of the liver	0.072 (0.016–0.178)
42	Gastric bleeding	0.21 (0.099–0.327)
43	Crohn’s disease or ulcerative colitis	0.136 (0.05–0.26)
44	Benign prostatic hypertrophy: symptomatic	0.05 (0.009–0.141)
45	Urinary incontinence	0.17 (0.074–0.282)
46	Stress incontinence	0.022 (0.002–0.084)
47	Impotence	0.016 (0.001–0.065)
	Infertility	
48	Primary	0.013 (0.001–0.059)
49	Secondary	0.011 (0.001–0.053)
50	Heart burn & reflux “GERD”	0.062 (0.012–0.162)
	Chronic respiratory disease	
	Asthma	
51	Controlled	0.022 (0.002–0.084)
52	Partially controlled	0.045 (0.007–0.128)
53	Uncontrolled	0.221 (0.105–0.34)
	Chronic obstructive pulmonary disease (COPD) and other chronic respiratory diseases	
54	Mild	0.017 (0.001–0.069)
55	Moderate	0.175 (0.074–0.294)
56	Severe	0.265 (0.142–0.379)
	Neurological disorders	
	Dementia	
57	Mild	0.027 (0.003–0.096)
58	Moderate	0.132 (0.045–0.254)
59	Severe	0.243 (0.126–0.359)
	Headache	
60	Migraine	0.534 (0.426–0.649)
61	Tension-type	0.109 (0.034–0.227)
62	Medication overuse	0.205 (0.093–0.33)
	Multiple sclerosis	
63	Mild	0.124 (0.042–0.242)
64	Moderate	0.315 (0.195–0.418)
65	Severe	0.689 (0.578–0.81)
	Epilepsy	
66	Severe (seizures > = once a month)	0.57 (0.467–0.687)
67	Less severe (seizures 1–11 per year)	0.425 (0.305–0.528)
	Parkinson’s disease	
68	Mild	0.025 (0.002–0.092)
69	Moderate	0.333 (0.209–0.436)
70	Severe	0.559 (0.448–0.682)
	Mental, behavioural, and substance use disorders	
	Alcohol use disorder	
71	Very mild	0.033 (0.004–0.111)
72	Mild	0.127 (0.045–0.247)
73	Moderate	0.23 (0.115–0.349)
74	Severe	0.286 (0.169–0.396)
	Fetal alcohol syndrome	
75	Mild	0.028 (0.003–0.098)
76	Moderate	0.07 (0.016–0.173)
77	Severe	0.132 (0.047–0.247)
	Cannabis dependence	
78	Mild	0.279 (0.166–0.386)
79	Severe	0.474 (0.361–0.585)
	Amphetamine dependence	
80	Mild	0.266 (0.144–0.379)
81	Severe	0.613 (0.502–0.729)
	Cocaine dependence	
82	Mild	0.24 (0.127–0.355)
83	Severe	0.534 (0.422–0.652)
	Heroin and other opioid dependence	
84	Mild	0.428 (0.318–0.528)
85	Severe	0.699 (0.579–0.827)
	Anxiety disorders	
86	Mild	0.024 (0.002–0.091)
87	Moderate	0.113 (0.035–0.233)
88	Severe	0.49 (0.375–0.603)
	Major depressive disorder	
89	Mild episode	0.067 (0.015–0.171)
90	Moderate episode	0.509 (0.404–0.617)
91	Severe episode	0.607 (0.497–0.731)
	Bipolar disorder	
92	Manic episode	0.506 (0.398–0.616)
93	Residual state	0.035 (0.005–0.109)
	Schizophrenia	
94	Acute state	0.69 (0.583–0.818)
95	Residual state	0.458 (0.341–0.569)
96	Anorexia nervosa	0.072 (0.017–0.176)
97	Bulimia nervosa	0.048 (0.008–0.139)
98	Attention deficit hyperactivity disorder	0.019 (0.002–0.074)
99	Conduct disorder	0.196 (0.09–0.315)
100	Borderline intellectual functioning	0.015 (0.001–0.063)
	Intellectual disability/mental retardation	
101	Mild	0.056 (0.011–0.157)
102	Moderate	0.084 (0.022–0.195)
103	Severe	0.085 (0.023–0.196)
104	Profound	0.189 (0.085–0.307)
	Hearing and vision loss	
	Hearing loss	
105	Mild	0.021 (0.002–0.079)
106	Moderate	0.053 (0.009–0.147)
107	Severe	0.219 (0.108–0.338)
108	Profound	0.205 (0.096–0.329)
109	Complete	0.164 (0.066–0.279)
110	Mild, with ringing	0.032 (0.004–0.107)
111	Moderate, with ringing	0.058 (0.011–0.153)
112	Severe, with ringing	0.29 (0.172–0.402)
113	Profound, with ringing	0.201 (0.096–0.32)
114	Complete, with ringing	0.298 (0.173–0.408)
	Distance vision	
115	Mild impairment	0.008 (0–0.041)
116	Moderate impairment	0.023 (0.002–0.087)
117	Severe impairment	0.232 (0.123–0.346)
118	Blindness	0.194 (0.087–0.313)
119	Monocular	0.032 (0.004–0.106)
120	Presbyopia	0.009 (0–0.045)
	Musculoskeletal disorders	
	Low back pain	
121	Mild	0.026 (0.003–0.093)
122	Moderate	0.081 (0.021–0.192)
123	Severe, without leg pain	0.141 (0.053–0.258)
124	Severe, with leg pain	0.151 (0.059–0.269)
125	Most severe, without leg pain	0.197 (0.089–0.313)
126	Most severe, with leg pain	0.207 (0.099–0.326)
	Neck pain	
127	Mild	0.024 (0.003–0.089)
128	Moderate	0.064 (0.013–0.162)
129	Severe	0.133 (0.047–0.254)
130	Most severe	0.119 (0.039–0.235)
	Musculoskeletal problems	
131	Legs, mild	0.031 (0.004–0.102)
132	Legs, moderate	0.111 (0.035–0.226)
133	Legs, severe	0.147 (0.057–0.269)
134	Arms, mild	0.025 (0.002–0.088)
135	Arms, moderate	0.088 (0.023–0.196)
136	Generalized, moderate	0.147 (0.054–0.264)
137	Generalized, severe	0.335 (0.214–0.436)
138	Gout, acute	0.184 (0.08–0.305)
	Injury	
139	Amputation of one upper limb (long term, without treatment)	0.133 (0.05–0.255)
140	Concussion (short term)	0.048 (0.008–0.138)
141	Spinal cord lesion, below neck level (treated)	0.399 (0.27–0.501)
	Other	
	Abdominopelvic problem	
142	Mild	0.03 (0.003–0.107)
143	Moderate	0.105 (0.033–0.221)
144	Severe	0.334 (0.222–0.432)
	Anaemia	
145	Mild	0.005 (0–0.027)
146	Moderate	0.053 (0.01–0.145)
147	Severe	0.146 (0.057–0.266)
148	Periodontitis	0.008 (0–0.038)
149	Dental caries: symptomatic	0.009 (0–0.044)
150	Severe tooth loss	0.031 (0.004–0.103)
	Disfigurement	
151	Level 1	0.034 (0.005–0.114)
152	Level 2	0.131 (0.046–0.251)
153	Level 3	0.628 (0.52–0.751)
154	Level 1, with itch or pain	0.053 (0.01–0.142)
155	Level 2, with itch or pain	0.192 (0.086–0.313)
156	Level 3, with itch or pain	0.641 (0.536–0.763)
	Generic uncomplicated disease	
157	Worry and daily medication	0.022 (0.002–0.082)
158	Anxiety about diagnosis	0.007 (0–0.035)
159	Kwashiorkor	0.05 (0.009–0.146)
160	Severe wasting	0.079 (0.018–0.186)
161	Speech problems	0.021 (0.002–0.077)
	Motor impairment	
162	Mild	0.016 (0.001–0.07)
163	Moderate	0.041 (0.007–0.124)
164	Severe	0.168 (0.068–0.295)
	Motor plus cognitive impairments	
165	Mild	0.041 (0.006–0.128)
166	Moderate	0.07 (0.014–0.179)
167	Severe	0.252 (0.139–0.359)
168	Rectovaginal fistula	0.432 (0.311–0.539)
169	Vesicovaginal fistula	0.211 (0.102–0.323)
170	Thrombocytopenic purpura	0.09 (0.024–0.203)
171	Hypothyroidism	0.012 (0.001–0.054)
172	Hyperthyroidism	0.05 (0.008–0.138)
173	Neck pain, moderate	0.078 (0.02–0.188)
174	Osteomyelitis	0.074 (0.017–0.181)
175	Shoulder lesions	0.013 (0.001–0.057)
176	Heart burn & reflux “GERD”	0.062 (0.013–0.174)
177	Constipation	0.032 (0.004–0.108)
178	Vaginal discharge	0.009 (0–0.047)
179	Dyspareunia	0.009 (0–0.045)
180	Stress incontinence	0.021 (0.002–0.081)
181	Irritable bowel syndrome	0.038 (0.005–0.118)
182	Somatoform disorder	0.089 (0.023–0.199)
183	Borderline personality disorder	0.167 (0.071–0.287)
184	Harmful alcohol use	0.068 (0.016–0.168)
185	Vertigo and balance disorder (Menière, labyrinthitis)	0.044 (0.007–0.131)
186	Trigeminal neuralgia	0.053 (0.009–0.142)
187	Encephalopathy—moderate	0.142 (0.052–0.259)
188	Encephalopathy—severe	0.274 (0.155–0.383)
189	Thrombocytopenic purpura	0.074 (0.018–0.183)
190	Lymphogranuloma Venereum—local infection	0.053 (0.01–0.144)
191	Subacute sclerosing panencephalitis—phase 1	0.039 (0.006–0.121)
192	Subacute sclerosing panencephalitis—phase 2	0.102 (0.029–0.219)
193	Subacute sclerosing panencephalitis—phase 3	–
194	Haemorrhoids	0.049 (0.009–0.143)
195	Anal fissure/abcess/fistula	0.039 (0.005–0.122)
196	Hyperthyroidism	0.05 (0.008–0.141)
197	Allergic rhinitis (hay fever)	0.005 (0–0.029)
198	Varicose veins	0.024 (0.002–0.088)
199	Carpal tunnel syndrome	0.019 (0.002–0.074)
200	Intensive care unit admission	0.547 (0.429–0.653)
201	Invasive device/drain	0.103 (0.031–0.216)
202	Insomnia	0.019 (0.002–0.076)
203	Sleep apnoea	0.05 (0.008–0.14)
204	Hypothyroidism	0.019 (0.002–0.074)
205	Hearing loss, moderate (modified)	0.069 (0.016–0.176)
206	Hearing loss, severe (modified)	0.297 (0.177–0.403)

**Table 3:** Distribution of disability weights for the 206 health states

Disability weight	GBD 2013 N (%)	The Wuhan survey* N (%)
0.0–0.1	89 (43.2%)	108 (52.4%)
0.1–0.2	41 (19.9%)	40 (19.4%)
0.2–0.3	26 (12.6%)	26 (12.6%)
0.3–0.4	15 (7.3%)	9 (4.4%)
0.4–0.5	14 (6.8%)	6 (2.9%)
0.5–0.6	16 (7.8%)	9 (4.4%)
0.6–0.7	3 (1.5%)	7 (3.4%)
0.7–0.8	2 (1.0%)	0

*There were 205 DW values because the value of health state 'Subacute sclerosing panencephalitis—phase 3' wasn’t available in Wuhan survey

For Wuhan population, health state “mild anemia” (DW = 0.005, 95% UI 0.000–0.027) or “allergic rhinitis (hay fever)” (0.005, 95% UI 0.000–0.029) had the lowest value and “Heroin and other opioid dependence, severe” had the highest value (DW = 0.699, 95% UI 0.579–0.827) (Table 2). These DW estimates were all statistically significant. The DW value of severe heroin dependence corresponding to mental, behavioural, and substance use disorders in Wuhan, indicating that patients with severe heroin dependence lose an average of more than two-thirds of a healthy life year for every one year of survival.

### Disease symptoms and DW differences between Wuhan’s and GBD 2013's DW

Generally, the Pearson’s *r* was 0.876 (*P* < 0.001) between the combined DW of these health states for GBD 2013 DW study and the current study.

There were differences in DW of health states across different surveys. Table 4 shows the results of the regression analysis by key symptoms mentioned in the lay descriptions. Eight symptoms of these 11 key symptoms may be driving these differences. Health states with mental symptom, fatigue, and the residual category of other physical symptoms were statistically significantly associated with a lower Wuhan’s DW than the GBD’s DW. Health states with disfigurement and substance use symptom had higher Wuhan’s DW than the GBD’s DW, with significantly statistical difference.

**Table 4:** Regression analysis results for proportional differences between the Wuhan’s DW and GBD’s DW for 206 comparable health states

Symptom (number of lay descriptions*)	Coefficient	95% confidence intervals	*P*
Mobility (26)	− 15.6	− 43.9 to 12.6	0.276
Pain (72)	− 19.9	− 41.7 to 1.94	0.074
Mental symptom (58)	− 39.1	− 60.8 to − 17.4	<0.001
Fatigue (50)	− 23.4	− 45.7 to − 1.0	0.040
Disfigurement (9)	70.2	24.0 to 116.3	0.003
Sensory symptom (21)	24.0	− 11.0 to 58.9	0.177
Infection/diarrhoea (16)	− 20.9	− 56.7 to 15.0	0.252
Substance use (13)	56.8	17.6 to 96.0	0.005
ADL (83)	− 4.0	− 23.7 to 15.7	0.690
Cognitive symptom (30)	− 29.4	− 59.0 to 0.2	0.052
Others (75)	− 22.8	− 43.3 to − 2.4	0.028

*The number of lay descriptions add to more than the total because lay descriptions often combine several symptom categories

*DW* Disability weight; *ADL* Activities of daily living; *Others* Other physical symptoms, including dyspnoea, nausea, palpitations, reduced appetite, sleeping problems

## Discussion

The assessment of disease burden has been recommended to inform decision-making, which requires measuring the impact of disease on quality of life using DW [26–28]. The PC method could be used to estimate cause-specific DW for the calculation of DALY and health-adjusted life expectancy (HALE) [15]. In China, previous studies estimated cause-specific DW by asked health professionals to value health states [29], or by EQ-5D method [30]. The cause-specific DW accessed by the PC approaches has been lacking for regions of China. We performed this disability weight survey in Wuhan population by following GBD 2013 DW study.

We calculated and compared these findings in Wuhan population with GBD study. The set of DWs were bounded by health state “mild anemia” due to endocrine, metabolic, blood, and immune disorders or “allergic rhinitis (hay fever)” (DW = 0.005), and heroin dependence corresponding to mental and substance use disorders (DW = 0.699). This finding is inconsistent with GBD 2013 DW study which showed DWs ranged from 0.003 for mild distance vision impairment to 0.778 for acute schizophrenia [7]. We observed a higher correlation (Pearson’s* r* = 0.874) between DWs of same health states from Wuhan and the GBD 2013 study. This finding is also inconsistent with previous DW study in Asian country. In South Korea, the health state with the highest DW (0.912) was ‘‘Spinal cord lesion at neck level: untreated’’ and the lowest DW was ‘Distance vision mild impairment’ with 0.084 [5]. In Japanese DW study, the DWs of those health states from GBD 2013 study ranged from 0.707 for spinal cord injury at neck level (untreated) to 0.004 for mild anemia [25]. The differences in DW estimates were contributed to cultural differences which impact the ways people perceive health problems and how such problems affect their lives [20, 31, 32]. Our study showed the span of DW (0.005–0.699) in Wuhan, China was similar to that in the European DW measurement study (DW: 0.004–0.677) [6]. The findings suggest that there might be culture or contextual differences in perception of disease severity compared with different survey conducted elsewhere [19, 33]. These differences could have substantial implications for the magnitude or ranking of disease burdens. In this study, the set of DWs were more appropriate to the Wuhan population than GBD study, which could be used to quantify local disease burdens and suggested ranking of diseases.

Besides, age [34, 35], education level [36], and income level [37] might be the potential factors to access the severity of disability. Evenly, disease status might have an impact on DW estimates [36]. These factors should be specifically taken into the implications for DW estimates.

In this present study, the ranking of certain health states seems counterintuitive. Health state “Cancer: terminal phase, with medication” had a higher DW (0.568, 95% UI 0.462–0.690) than “Cancer: terminal phase, without medication” (0.344, 95% UI 0.223–0.445) which tend to be more severe. Severe, profound and complete hearing loss also showed this counterintuitive condition, as well as severe and profound hearing loss with ringing. Apart from that, severe and most severe neck pain also had this kind of situation. The underlying reasons of the inconsistencies might be related to the setting of the wording for lay descriptions of health states [6]. Brief lay descriptions were used to describe the major functional outcomes and symptoms associated with the health state, as reported in GBD 2013 and European DW study [6, 7]. The disease label was removed from the description to avoid elicit bias for stigmatizing conditions, which indicated the respondents didn’t know the cause of these health conditions. These types of findings need to be addressed with empirical investigation to understand whether the weights in question are sensitive to specific elements in the lay descriptions [7].

In summary, tackling the diverse challenges faced by local health-care systems is public policy priorities for China, as well as the quantification of localized disease burdens. This set of DWs could be used to calculate YLD, DALY and HALE caused by diseases for Wuhan, China. These changes of the severity of health state will require an integrated government response to improve primary care. Then, analysis of disease burden will provide a useful framework to guide policy responses to the changing disease spectrum in China. The DW measurement study in other region of China could be further researched.

### Limitations

There are some limitations in this study. Firstly, this study included participants aged 70 years or older in household survey, with approximately 9.4% (245) of 2610 respondents. This percent of the age group was 9.2% in the 2020 general population of Wuhan [38]. However, people aged over 70 were excluded in web survey. The old age may have impacted on valuation of the severity of health states. Thus, people aged over 70 could be included in the next study. Secondly, the DW differences were possibly attributed to variation between countries and alteration of the wording of lay descriptions of health state. Besides, the COVID-19 pandemic has given people the new insight and viewpoint to public health [39], and residents may exhibit greater risk perception of the pandemic [40, 41]. COVID-19 may cause bias in DW valuation due to the pandemic may cause people’s cognition and perception on health [42, 43]. Finally, we would make further efforts to increase the sample size from household survey in the next study.

## Conclusions

This study provided a set of DWs for Wuhan population. The DWs of these health states ranged from 0.005 for mild anemia or allergic rhinitis (hay fever) to 0.699 for severe heroin dependence. We found lower severity to mental and fatigue symptoms and higher severity to disfigurement and substance use symptoms in Wuhan’s DW study compared with GBD 2013 study. There might be contextual or culture differences that people have different perceptions of the severity of the disease across different surveys. A high correlation in DW of same health states was observed between Wuhan and the GBD 2013 study, and these DW estimates may be more appropriate for Wuhan population than GBD 2013, which could be used to the calculation of local diseases burden for health policy-decision. This study provides an empirical basis for DW survey in Hubei province and other regions of China.

## Supplementary Information

**Additional file 1: Table 1.** Pearson’s correlation coefficients between probit regression analyses for each survey and those from combined data. **Table 2.** Lay descriptions for 206 health states and symptom categories. GBD: Global Burden of Disease study; DW: Disability Weight; ADL: activities of daily living; Others: other physical symptoms, including dyspnoea, nausea, palpitations, reduced appetite, sleeping. **Table 3.** 28 health states used in population health equivalence method.

## Data Availability

The datasets used and/or analyzed during the current study are available from the corresponding author on reasonable request.

## Abbreviations

ADL: Activities of daily living
DALY: Disease-adjusted life years
DW: Disability weight
EQ-5D: European quality of life questionnaire
GBD: Global burden of disease
HALE: Health-adjusted life expectancy
PC: Paired comparison
PHE: Population health equivalence
UI: Uncertainty intervals
YLD: Years lived with disability
YLL: Years of life lost
