# Supplementary material for: Disability weight measurement for the severity of different diseases in Wuhan, China
Source: Popul Health Metr. 2023 May 4;21:5. doi: 10.1186/s12963-023-00304-y (PMC10157574; doi:10.1186/s12963-023-00304-y)
Supplement: Supplementary file 1 — Additional file 1: Table 1. Pearson’s correlation coefficients between probit regression analyses for each survey and those from combined data. Table 2. Lay descriptions for 206 health states and symptom categories. GBD: Global Burden of Disease study; DW: Disability Weight; ADL: activities of daily living; Others: other physical symptoms, including dyspnoea, nausea, palpitations, reduced appetite, sleeping. Table 3. 28 health states used in population health equivalence method. [file 12963_2023_304_MOESM1_ESM.docx]

**Paired comparison**

To examine measurement error and internal consistency of these consistent answers from participants, heat maps of response probabilities from PC questions were drafted. Figure 1 shows a heatmap of the PC response probabilities for the possible paired comparisons of 206 health states for Wuhan’s respondents. The horizontal coordinate represents the first health states in the paired comparison, and the vertical coordinate represents the second health states in the paired comparison. Each cell in the heat map indicates the response probability for one pair of health states. The colors of the heat map correspond to the probability that the first health state in a pair comparison is chosen as the healthier outcome. Very small amount of measurement error and high internal consistency were indicated by a completely smooth transition in colors from high to low probabilities between the upper left and lower right corner instead of a completely randomly color distribution. Figure 1 didn’t show a complete smooth transition from blue to red in Wuhan survey. This indicated there might be a small measurement error, or related to the sample size (see Appendix Figure 7: Response probabilities for paired comparisons for Chinese provinces with <5,000 respondents in subnational DW estimates)^1^. The number of participants from Wuhan’s survey should be increased to test the measurement error of PC method in the next study.

The respondents were given the same pair in the third, 10th and 16th PC questions. This deliberate repetition allows assessment of test-retest reliability of PC responses. In household survey, of 2610 participants who responded to the same pair of states in the repeated PC questions, 1777 (68%) of these respondents were consistent; in web-based survey, of 3140 participants who responded to the same pair of states in the third, 10th and16th PC questions, 78% (2461) of these participants were consistent. This percent is close to that from GBD 2013 DW study which reported that 71% of the total included respondents were consistent to the test-retest PC questions^2^.

Table 2 shows the linear correlations between the probit regression results on the PC responses for each survey and those from the combined data. In this study, comparison of the regression results showed high linear correlations between household and web-based survey (Pearson’s *r* = 0.857; *P* < 0.001); either of the two survey was highly correlated with the combined data (Pearson’s *r* > 0.9; *P* < 0.001).

**Appendix Table 1: Pearson’s correlation coefficients between probit regression analyses for each survey and those from combined data.**

|  | Household survey | Web-based survey | Combined survey |
| --- | --- | --- | --- |
| Household survey | - | 0.8380* | 0.9154* |
| Web-based survey |  | - | 0.9686* |

**P*-value<0.05

**Appendix table 2: Lay descriptions for 206 health states and symptom categories**^1^

| id | Sequela | Health state | Lay description | DW in previous GBD study | Symptoms | | | | | | | | | | |
| --- | --- | --- | --- | --- | --- | --- | --- | --- | --- | --- | --- | --- | --- | --- | --- |
|  |  |  |  |  | Mobility | Pain | Mental symptom | Fatigue | Disfigurement | Sensory symptom | Infection/  diarrhea | Substance  use | ADL | Cognition | Others |
|  | **Infectious disease** |  |  |  |  |  |  |  |  |  |  |  |  |  |  |
| 1 | Mild other oral disorders | Infectious disease, acute episode, mild | has a low fever and mild discomfort, but no difficulty with daily activities. | 0.006 (0.002 0.012) |  |  |  |  |  |  | ○ |  |  |  |  |
| 2 | Severe other oral disorders | Infectious disease, acute episode, moderate | has a fever and aches, and feels weak, which causes some difficulty with daily activities. | 0.051 (0.032 0.074) |  | ○ |  | ○ |  |  | ○ |  | ○ |  |  |
| 3 | Severe cellulitis | Infectious disease, acute episode, severe | has a high fever and pain, and feels very weak, which causes great difficulty with daily activities. | 0.133 (0.088 0.190) |  | ○ |  | ○ |  |  | ○ |  | ○ |  |  |
| 4 | Post Ebola chronic fatigue syndrome | Infectious disease, post-acute consequences (fatigue, emotional lability, and insomnia) | is always tired and easily upset. The person feels pain all over the body and is depressed. | 0.219 (0.148 0.308) |  | ○ | ○ | ○ |  |  |  |  |  |  |  |
| 5 | Mild diarrhea due to schistosomiasis | Diarrhea, mild | has diarrhea three or more times a day with occasional discomfort in the belly. | 0.074 (0.049 0.104) |  |  |  |  |  |  | ○ |  |  |  |  |
| 6 | Moderate diarrheal diseases | Diarrhea, moderate | has diarrhea three or more times a day, with painful cramps in the belly and feeling thirsty. | 0.188 (0.125 0.264) |  | ○ |  |  |  |  | ○ |  |  |  |  |
| 7 | Severe diarrheal diseases | Diarrhea, severe | has diarrhea three or more times a day with severe belly cramps. The person is very thirsty and feels nauseous and tired. | 0.247 (0.164 0.348) |  | ○ |  | ○ |  |  | ○ |  |  |  |  |
| 8 | Hydrocele due to lymphatic filariasis | Epididymo-orchitis | has swelling and tenderness in the testicles and pain during urination. | 0.128 (0.086 0.180) |  | ○ |  |  |  |  | ○ |  |  |  |  |
| 9 | Herpes zoster | Herpes zoster | has a blistering skin rash that causes pain, with some burning and itching. | 0.058 (0.035 0.090) |  | ○ |  |  |  |  |  |  |  |  |  |
| 10 | Symptomatic HIV without anemia | HIV: symptomatic, pre-AIDS | has weight loss, fatigue, and frequent infections. | 0.274 (0.184 0.377) |  | ○ |  | ○ |  |  | ○ |  |  |  |  |
| 11 | HIVIAIDS with antiretroviral treatment without anemia | HIV/AIDS: receiving antiretroviral (ARV) treatment | has occasional fevers and infections. The person takes daily medication that sometimes causes diarrhea. | 0.078 (0.052 0.111) |  | ○ |  |  |  |  | ○ |  |  |  |  |
| 12 | AIDS without anemia | AIDS: not receiving antiretroviral (ARV) treatment | has severe weight loss, weakness, fatigue, cough and fever, and frequent infections, skin rashes and diarrhea. | 0.582 (0.406 0.743) |  |  |  | ○ | ○ |  | ○ |  |  |  | ○ |
| 13 | Heavy infestation of hookworm | Intestinal nematode infections: symptomatic | has cramping pain and a bloated feeling in the belly. | 0.027 (0.015 0.043) |  | ○ |  |  |  |  |  |  |  |  |  |
| 14 | Lymphedema due to lymphatic filariasis | Lymphatic filariasis: symptomatic | has swollen legs with hard and thick skin, which causes difficulty in moving around. | 0.109 (0.073 0.154) | ○ |  |  |  |  |  |  |  |  |  |  |
| 15 | Severe infectious complications due to chronic otitis media | Ear pain | has an earache that causes some difficulty with daily activities. | 0.013 (0.007 0.024) |  | ○ |  |  |  |  |  |  | ○ |  |  |
| 16 | Extensively drug resistant tuberculosis | Tuberculosis, not HIV infected | has a persistent cough and fever, is short of breath, feels weak, and has lost a lot of weight. | 0.333 (0.224 0.454) |  |  |  |  |  |  | ○ |  |  |  |  |
| 17 | HIVIAIDS Extensively drug resistant Tuberculosis without anemia | Tuberculosis, HIV infected | has a persistent cough and fever, shortness of breath, night sweats, weakness and fatigue and severe weight loss. | 0.408 (0.274 0.549) |  |  |  | ○ |  |  | ○ |  |  |  |  |
|  | **Cancer** |  |  |  |  |  |  |  |  |  |  |  |  |  |  |
| 18 | Diagnosis and primary therapy phase of other malignant neoplasms | Cancer, diagnosis and primary therapy | has pain, nausea, fatigue, weight loss and high anxiety. | 0.288 (0.193 0.399) |  | ○ | ○ | ○ |  |  |  |  |  |  |  |
| 19 | Metastatic phase of other malignant neoplasms | Cancer, metastatic | has severe pain, extreme fatigue, weight loss and high anxiety. | 0.451 (0.307 0.600) |  | ○ | ○ | ○ |  |  |  |  |  |  |  |
| 20 | Terminal phase of other malignant neoplasms | Terminal phase, with medication (for cancers and end-stage kidney or liver disease) | has lost a lot of weight and regularly uses strong medication to avoid constant pain. The person has no appetite, feels nauseous, and needs to spend most of the day in bed. | 0.540 (0.377 0.687) |  | ○ |  | ○ |  |  |  |  | ○ |  |  |
| 21 | Stage V chronic kidney disease untreated without anemia due to other causes | Terminal phase, without medication (for cancers and end-stage kidney or liver disease) | has lost a lot of weight and has constant pain. The person has no appetite, feels nauseous, and needs to spend most of the day in bed. | 0.569 (0.389 0.727) |  | ○ |  | ○ |  |  |  |  | ○ |  |  |
|  | **Cardiovascular and circulatory disease** |  |  |  |  |  |  |  |  |  |  |  |  |  |  |
| 22 | Acute myocardial infarction first to 2 days | Acute myocardial infarction, days 1-2 | has severe chest pain that becomes worse with any physical activity. The person feels nauseous, short of breath, and very anxious. | 0.432 (0.288 0.579) |  | ○ |  |  |  |  |  |  |  |  | ○ |
| 23 | Acute myocardial infarction 3 to 28 days | Acute myocardial infarction, days 3-28 | gets short of breath after heavy physical activity, and tires easily, but has no problems when at rest. The person has to take medication every day and has some anxiety. | 0.074 (0.049 0.105) |  |  | ○ | ○ |  |  |  |  |  |  | ○ |
| 24 | Mild angina due to ischemic heart disease | Angina pectoris, mild | has chest pain that occurs with strenuous physical activity, such as running or lifting heavy objects. After a brief rest, the pain goes away. | 0.033 (0.020 0.052) |  | ○ |  |  |  |  |  |  |  |  |  |
| 25 | Moderate angina due to ischemic heart disease | Angina pectoris, moderate | has chest pain that occurs with moderate physical activity, such as walking uphill or more than half a kilometer (around a quarter mile) on level ground. After a brief rest, the pain goes away. | 0.080 (0.052 0.113) |  | ○ |  |  |  |  |  |  |  |  |  |
| 26 | Severe angina due to ischemic heart disease | Angina pectoris, severe | has chest pain that occurs with minimal physical activity, such as walking only a short distance. After a brief rest, the pain goes away. The person avoids most physical activities because of the pain. | 0.167 (0.110 0.240) |  | ○ |  |  |  |  |  |  | ○ |  |  |
| 27 | Symptomatic atrial fibrillation and flutter | Cardiac conduction disorders and cardiac dysrhythmias | has periods of rapid and irregular heartbeats and occasional fainting. | 0.224 (0.151 0.312) |  |  |  |  |  |  |  |  |  |  | ○ |
| 28 | Symptomatic claudication due to peripheral vascular disease | Claudication | has cramping pains in the legs after walking a medium distance. The pain goes away after a short rest. | 0.014 (0.007 0.025) |  | ○ |  |  |  |  |  |  |  |  |  |
| 29 | Mild heart failure due to endocrine, metabolic, blood, and immune disorders | Heart failure, mild | is short of breath and easily tires with moderate physical activity, such as walking uphill or more than a quarter mile on level ground. The person feels comfortable at rest or during activities requiring less effort. | 0.041 (0.026 0.062) |  |  |  | ○ |  |  |  |  | ○ |  | ○ |
| 30 | Moderate heart failure due to thalassemias | Heart failure, moderate | is short of breath and easily tires with minimal physical activity, such as walking only a short distance. The person feels comfortable at rest but avoids moderate activity. | 0.072 (0.047 0.103) |  |  |  | ○ |  |  |  |  | ○ |  | ○ |
| 31 | Severe heart failure due toendocrine, metabolic, blood, and immune disorders | Heart failure, severe | is short of breath and feels tired when at rest. The person avoids any physical activity, for fear of worsening the breathing problems. | 0.179 (0.122 0.251) |  |  |  | ○ |  |  |  |  | ○ |  | ○ |
| 32 | Chronic subarachnoid hemorrhage severity level 1 | Stroke, long term consequences, mild | has some difficulty in moving around and some weakness in one hand, but is able to walk without help. | 0.019 (0.010 0.032) |  |  |  | ○ |  |  |  |  |  |  |  |
| 33 | Chronic subarachnoid hemorrhage severity level 2 | Stroke, long term consequences, moderate | has some difficulty in moving around, and in using the hands for lifting and holding things, dressing and grooming. | 0.070 (0.046 0.099) |  |  |  | ○ |  |  |  |  |  |  |  |
| 34 | Chronic subarachnoid hemorrhage severity level 3 | Stroke, long term consequences, moderate plus cognition problems | has some difficulty in moving around, in using the hands for lifting and holding things, dressing and grooming, and in speaking. The person is often forgetful and confused. | 0.316 (0.206 0.437) |  |  |  | ○ |  |  |  |  |  | ○ |  |
| 35 | Chronic subarachnoid hemorrhage severity level 4 | Stroke, long term consequences, severe | is confined to bed or a wheelchair, has difficulty speaking and depends on others for feeding, toileting and dressing. | 0.552 (0.377 0.707) |  |  |  | ○ |  |  |  |  | ○ |  |  |
| 36 | Chronic subarachnoid hemorrhage severity level 5 | Stroke, long term consequences, severe plus cognition problems | is confined to bed or a wheelchair, depends on others for feeding, toileting and dressing, and has difficulty speaking, thinking clearly and remembering things. | 0.588 (0.411 0.744) |  |  |  | ○ |  |  |  |  | ○ | ○ |  |
|  | **Diabetes and digestive and genitourinary disease** |  |  |  |  |  |  |  |  |  |  |  |  |  |  |
| 37 | Diabetic neuropathy due to diabetes mellitus type 2 | Diabetic neuropathy | has pain, tingling and numbness in the arms, legs, hands and feet. The person sometimes gets cramps and muscle weakness. | 0.133 (0.089 0.187) | ○ | ○ |  |  |  |  |  |  |  |  |  |
| 38 | Stage IV chronic kidney disease untreated without anemia due to other causes | Chronic kidney disease (stage IV) | tires easily, has nausea, reduced appetite and difficulty sleeping. | 0.104 (0.070 0.147) |  |  |  | ○ |  |  |  |  |  |  | ○ |
| 39 | End stage renal disease after transplant due to other causes | End-stage renal disease, with kidney transplant | sometimes feels tired and down, and has some difficulty with daily activities. | 0.024 (0.014 0.039) |  |  |  | ○ |  |  |  |  | ○ |  |  |
| 40 | End stage renal disease on dialysis due to other causes | End-stage renal disease, on dialysis | is tired and has itching, cramps, headache, joint pains and shortness of breath. The person needs intensive medical care every other day lasting about half a day. | 0.571 (0.398 0.725) |  | ○ |  | ○ |  |  |  |  | ○ |  | ○ |
| 41 | Cirrhosis and other chronic liver diseases due to other cause, decompensated | Decompensated cirrhosis of the liver | has a swollen belly and swollen legs. The person feels weakness, fatigue and loss of appetite. | 0.178 (0.123 0.250) |  |  |  | ○ |  |  |  |  |  |  | ○ |
| 42 | Hematemesis due to schistosomiasis | Gastric bleeding | vomits blood and feels nauseous. | 0.325 (0.209 0.462) |  |  |  |  |  |  |  |  |  |  | ○ |
| 43 | Ulcerative colitis with moderate anemia | Crohn's disease or ulcerative colitis | has cramping abdominal pain, has diarrhea several times a day, and feels very tired for two months every year. When the person does not have symptoms, there is anxiety about them returning. | 0.231 (0.156 0.320) |  | ○ |  |  |  |  | ○ |  |  |  |  |
| 44 | Symptomatic benign prostatic hyperplasia | Benign prostatic hypertrophy: symptomatic | feels the urge to urinate frequently, but when passing urine it comes out slowly and sometimes is painful. | 0.067 (0.043 0.097) |  | ○ |  |  |  |  |  |  |  |  | ○ |
| 45 | Incontinence due to congenital anomalies of the urinary tract | Urinary incontinence | cannot control urinating. | 0.139 (0.094 0.198) |  |  |  |  |  |  |  |  |  |  | ○ |
| 46 | Stress incontinence due to genital prolapse | Stress incontinence | loses small amounts of urine without meaning to when coughing, sneezing, laughing or during physical exercise. | 0.020 (0.011 0.035) |  |  |  |  |  |  |  |  |  |  | ○ |
| 47 | Impotence due to congenital genital anomalies | Impotence | has difficulty in obtaining or maintaining an erection. | 0.017 (0.009 0.030) |  |  |  |  |  |  |  |  |  |  | ○ |
| 48 | Primary infertility due to endometriosis | Infertility, primary | wants to have a child and has a fertile partner, but the couple cannot conceive. | 0.008 (0.003 0.015) |  |  |  |  |  |  |  |  |  |  | ○ |
| 49 | Secondary infertility due to endometriosis | Infertility, secondary | has at least one child, and wants to have more children. The person has a fertile partner, but the couple cannot conceive. | 0.005 (0.002 0.011) |  |  |  |  |  |  |  |  |  |  | ○ |
| 50 | Severe or very severe gastroesophageal reflux disease symptoms (typical) | Heart burn & reflux “GERD” | Often has a burning sensation in the back of the chest after eating. | 0.026 (0.015 0.042) |  | ○ |  |  |  |  |  |  |  |  |  |
|  | **Chronic respiratory disease** |  |  |  |  |  |  |  |  |  |  |  |  |  |  |
| 51 | Controlled asthma | Asthma, controlled | has wheezing and cough once a month, which does not cause difficulty with daily activities. | 0.015 (0.007 0.026) |  |  |  |  |  |  |  |  |  |  | ○ |
| 52 | Partially controlled asthma | Asthma, partially controlled | has wheezing and cough once a week, which causes some difficulty with daily activities. | 0.036 (0.022 0.055) |  |  |  |  |  |  |  |  | ○ |  | ○ |
| 53 | Uncontrolled asthma | Asthma, uncontrolled | has wheezing, cough and shortness of breath more than twice a week, which causes difficulty with daily activities and sometimes wakes the person at night. | 0.133 (0.086 0.192) |  |  |  |  |  |  |  |  | ○ |  | ○ |
| 54 | Mild chronic respiratory problems and breathlessness due to congenital diaphragmatic hernia | Chronic obstructive pulmonary disease (COPD) and other chronic respiratory problems, mild | has cough and shortness of breath after heavy physical activity, but is able to walk long distances and climb stairs. | 0.019 (0.011 0.033) |  |  |  |  |  |  |  |  |  |  | ○ |
| 55 | Moderate respiratory problems due to motor neuron disease | COPD and other chronic respiratory problems, moderate | has cough, wheezing and shortness of breath, even after light physical activity. The person feels tired and can walk only short distances or climb only a few stairs. | 0.225 (0.153 0.310) |  |  |  | ○ |  |  |  |  | ○ |  | ○ |
| 56 | Severe interstitial lung disease and pulmonary sarcoidosis without heart failure | COPD and other chronic respiratory problems, severe | has cough, wheezing and shortness of breath all the time. The person has great difficulty walking even short distances or climbing any stairs, feels tired when at rest, and is anxious. | 0.408 (0.273 0.556) |  |  | ○ | ○ |  |  |  |  | ○ |  | ○ |
|  | **Neurological disorders** |  |  |  |  |  |  |  |  |  |  |  |  |  |  |
| 57 | Mild dementia due to other chromosomal abnormalities | Dementia, mild | has some trouble remembering recent events, and finds it hard to concentrate and make decisions and plans. | 0.069 (0.046 0.099) |  |  |  |  |  |  |  |  | ○ | ○ |  |
| 58 | Moderate dementia due to other chromosomal abnormalities | Dementia, moderate | has memory problems and confusion, feels disoriented, at times hears voices that are not real, and needs help with some daily activities. | 0.377 (0.252 0.508) |  |  | ○ |  |  |  |  |  | ○ | ○ |  |
| 59 | Severe dementia due to other chromosomal abnormalities | Dementia, severe | has complete memory loss; no longer recognizes close family members; and requires help with all daily activities. | 0.449 (0.304 0.595) |  |  |  |  |  |  |  |  | ○ | ○ |  |
| 60 | Symptomatic migraine | Headache, migraine | has severe, throbbing head pain and nausea that cause great difficulty in daily activities and sometimes confine the person to bed. Moving around, light, and noise make it worse. | 0.441 (0.294 0.588) |  | ○ |  |  |  |  |  |  | ○ |  |  |
| 61 | Symptomatic tension type headache | Headache, tension-type | has a moderate headache that also affects the neck, which causes difficulty in daily activities. | 0.037 (0.022 0.057) |  | ○ |  |  |  |  |  |  | ○ |  |  |
| 62 | Symptomatic medication overuse headache due to tension type headache | Headache, medication overuse | has daily headaches, felt as dull pain and often lasting all day, with poor sleep, nausea and fatigue. The person takes medicine for the headaches, which provides little relief but is needed to avoid having worse symptoms. | 0.223 (0.146 0.313) |  | ○ |  | ○ |  |  |  |  |  |  | ○ |
| 63 | Mild multiple sclerosis | Multiple sclerosis, mild | has mild loss of feeling in one hand, is a little unsteady while walking, has slight loss of vision in one eye, and often needs to urinate urgently. | 0.183 (0.124 0.253) | ○ |  |  |  |  | ○ |  |  |  |  | ○ |
| 64 | Moderate multiple sclerosis | Multiple sclerosis, moderate | needs help walking, has difficulty with writing and arm coordination, has loss of vision in one eye and cannot control urinating. | 0.463 (0.313 0.613) | ○ |  |  | ○ |  |  |  |  |  |  | ○ |
| 65 | Severe multiple sclerosis | Multiple sclerosis, severe | has slurred speech and difficulty swallowing. The person has weak arms and hands, very limited and stiff leg movement, has loss of vision in both eyes and cannot control urinating. | 0.719 (0.534 0.858) | ○ |  |  | ○ |  | ○ |  |  |  |  | ○ |
| 66 | Idiopathic, severe epilepsy | Epilepsy, seizures >= once a month | has sudden seizures one or more times each month, with violent muscle contractions and stiffness, loss of consciousness, and loss of urine or bowel control. Between seizures the person has memory loss and difficulty concentrating. | 0.552 (0.375 0.710) |  |  |  |  |  |  |  |  |  | ○ | ○ |
| 67 | Idiopathic, less severe epilepsy | Epilepsy, seizures 1-11 per year | has sudden seizures two to five times a year, with violent muscle contractions and stiffness, loss of consciousness, and loss of urine or bowel control. | 0.263 (0.173 0.367) |  |  |  |  |  |  |  |  |  |  |  |
| 68 | Mild Parkinson disease | Parkinson disease, mild | has mild tremors and moves a little slowly, but is able to walk and do daily activities without assistance. | 0.010 (0.005 0.019) | ○ |  |  |  |  |  |  |  |  |  |  |
| 69 | Moderate Parkinson disease | Parkinson disease, moderate | has moderate tremors and moves slowly, which causes some difficulty in walking and daily activities. The person has some trouble swallowing, talking, sleeping, and remembering things. | 0.267 (0.181 0.372) | ○ |  |  |  |  |  |  |  | ○ | ○ | ○ |
| 70 | Severe Parkinson disease | Parkinson disease, severe | has severe tremors and moves very slowly, which causes great difficulty in walking and daily activities. The person falls easily and has a lot of difficulty talking, swallowing, sleeping, and remembering things. | 0.575 (0.396 0.730) | ○ |  |  |  |  |  |  |  | ○ | ○ | ○ |
|  | **Mental, behavioural, and substance use disorders** |  |  |  |  |  |  |  |  |  |  |  |  |  |  |
| 71 | Very mild alcohol dependence | Alcohol use disorder, very mild | drinks alcohol daily and has difficulty controlling the urge to drink. When sober, the person functions normally. | 0.123 (0.082 0.177) |  |  |  |  |  |  |  | ○ |  |  |  |
| 72 | Mild alcohol dependence | Alcohol use disorder, mild | drinks a lot of alcohol and sometimes has difficulty controlling the urge to drink. While intoxicated, the person has difficulty performing daily activities. | 0.235 (0.160 0.327) |  |  |  |  |  |  |  | ○ | ○ |  |  |
| 73 | Moderate alcohol dependence | Alcohol use disorder, moderate | drinks a lot, gets drunk almost every week and has great difficulty controlling the urge to drink. Drinking and recovering cause great difficulty in daily activities, sleep loss, and fatigue. | 0.373 (0.248 0.508) |  |  |  | ○ |  |  |  | ○ | ○ |  | ○ |
| 74 | Severe alcohol dependence | Alcohol use disorder, severe | gets drunk almost every day and is unable to control the urge to drink. Drinking and recovering replace most daily activities. The person has difficulty thinking, remembering and communicating, and feels constant pain and fatigue. | 0.570 (0.396 0.732) |  | ○ |  | ○ |  |  |  | ○ |  | ○ |  |
| 75 | Mild fetal alcohol syndrome | Fetal alcohol syndrome, mild | is a little slow in developing physically and mentally, which causes some difficulty in learning but no other difficulties in daily activities. | 0.016 (0.008 0.030) |  |  |  |  |  |  |  |  |  | ○ |  |
| 76 | Moderate fetal alcohol syndrome | Fetal alcohol syndrome, moderate | is slow in developing physically and mentally, which causes some difficulty in daily activities. | 0.056 (0.035 0.083) |  |  |  |  |  |  |  |  | ○ | ○ |  |
| 77 | Severe fetal alcohol syndrome | Fetal alcohol syndrome, severe | is very slow in developing physically and mentally, which causes great difficulty in daily activities. | 0.179 (0.119 0.257) |  |  |  |  |  |  |  |  | ○ | ○ |  |
| 78 | Mild cannabis dependence | Cannabis dependence, mild | uses marijuana at least once a week and has some difficulty controlling the habit. When not using, the person functions normally. | 0.039 (0.024 0.060) |  |  |  |  |  |  |  | ○ |  |  |  |
| 79 | Severe cannabis dependence | Cannabis dependence, severe | uses marijuana daily and has difficulty controlling the habit. The person sometimes has mood swings, anxiety and hallucinations, and has some difficulty in daily activities. | 0.266 (0.178 0.364) |  |  | ○ |  |  |  |  | ○ | ○ |  |  |
| 80 | Mild amphetamine dependence | Amphetamine dependence, mild | uses stimulants (drugs) at least once a week and has some difficulty controlling the habit. When not using, the person functions normally. | 0.079 (0.051 0.114) |  |  |  |  |  |  |  | ○ |  |  |  |
| 81 | Severe amphetamine dependence | Amphetamine dependence, severe | uses stimulants (drugs) and has difficulty controlling the habit. The person sometimes has depression, hallucinations and mood swings, and has difficulty in daily activities. | 0.486 (0.329 0.637) |  |  | ○ |  |  |  |  | ○ | ○ |  |  |
| 82 | Other drug use disorders | Cocaine dependence, mild | uses cocaine at least once a week and has some difficulty controlling the habit. When not using, the person functions normally. | 0.116 (0.074 0.165) |  |  |  |  |  |  |  | ○ |  |  |  |
| 83 | Severe cocaine dependence | Cocaine dependence, severe | uses cocaine and has difficulty controlling the habit. The person sometimes has mood swings, anxiety, paranoia, hallucinations and sleep problems, and has some difficulty in daily activities. | 0.479 (0.324 0.634) |  |  | ○ |  |  |  |  | ○ | ○ |  | ○ |
| 84 | Mild opioid dependence | Heroin and other opioid dependence, mild | uses heroin (or methadone) a daily and has difficulty controlling the habit. When not using, the person functions normally. | 0.335 (0.221 0.473) |  |  |  |  |  |  |  | ○ |  |  |  |
| 85 | Severe opioid dependence | Heroin and other opioid dependence, severe | uses heroin daily and has difficulty controlling the habit. When the effects wear off, the person feels severe nausea, agitation, vomiting and fever. The person has a lot of difficulty in daily activities. | 0.697 (0.510 0.843) |  |  |  |  |  |  | ○ | ○ | ○ |  | ○ |
| 86 | Mild other mental disorders | Anxiety disorders, mild | feels mildly anxious and worried, which makes it slightly difficult to concentrate, remember things, and sleep. The person tires easily but is able to perform daily activities. | 0.030 (0.018 0.046) |  |  | ○ | ○ |  |  |  |  |  | ○ |  |
| 87 | Moderate other mental disorders | Anxiety disorders, moderate | feels anxious and worried, which makes it difficult to concentrate, remember things, and sleep. The person tires easily and finds it difficult to perform daily activities. | 0.133 (0.091 0.186) |  |  | ○ | ○ |  |  |  |  | ○ | ○ |  |
| 88 | Severe other mental disorders | Anxiety disorders, severe | constantly feels very anxious and worried, which makes it difficult to concentrate, remember things and sleep. The person has lost pleasure in life and thinks about suicide. | 0.523 (0.362 0.677) |  |  | ○ | ○ |  |  |  |  | ○ | ○ |  |
| 89 | Depression due to premenstrual syndrome | Major depressive disorder, mild episode | feels persistent sadness and has lost interest in usual activities. The person sometimes sleeps badly, feels tired, or has trouble concentrating but still manages to function in daily life with extra effort. | 0.145 (0.099 0.209) |  |  | ○ | ○ |  |  |  |  |  |  | ○ |
| 90 | Moderate major depressive disorder | Major depressive disorder, moderate episode | has constant sadness and has lost interest in usual activities. The person has some difficulty in daily life, sleeps badly, has trouble concentrating, and sometimes thinks about harming himself (or herself). | 0.396 (0.267 0.531) |  |  | ○ | ○ |  |  |  |  | ○ |  | ○ |
| 91 | Severe major depressive disorder | Major depressive disorder, severe episode | has overwhelming, constant sadness and cannot function in daily life. The person sometimes loses touch with reality and wants to harm or kill himself (or herself). | 0.658 (0.477 0.807) |  |  | ○ | ○ |  |  |  |  | ○ |  | ○ |
| 92 | Bipolar disorder manic state | Bipolar disorder, manic episode | is hyperactive, hears and believes things that are not real, and engages in impulsive and aggressive behavior that endanger the person and others. | 0.492 (0.341 0.646) |  |  | ○ |  |  |  |  |  |  | ○ |  |
| 93 | Bipolar disorder residual state | Bipolar disorder, residual state | has mild mood swings, irritability and some difficulty with daily activities. | 0.032 (0.018 0.051) |  |  | ○ |  |  |  |  |  | ○ |  |  |
| 94 | Schizophrenia acute state | Schizophrenia, acute state | hears and sees things that are not real and is afraid, confused, and sometimes violent. The person has great difficulty with communication and daily activities, and sometimes wants to harm or kill himself (or herself). | 0.778 (0.606 0.900) |  |  | ○ |  |  |  |  |  | ○ | ○ |  |
| 95 | Schizophrenia residual state | Schizophrenia, residual state | hears and sees things that are not real and has trouble communicating. The person can be forgetful, has difficulty with daily activities, and thinks about hurting himself (or herself). | 0.588 (0.411 0.754) |  |  | ○ |  |  |  |  |  | ○ | ○ |  |
| 96 | Anorexia nervosa | Anorexia nervosa | feels an overwhelming need to starve and exercises excessively to lose weight. The person is very thin, weak and anxious. | 0.224 (0.150 0.312) |  |  | ○ | ○ |  |  |  |  |  |  |  |
| 97 | Bulimia nervosa | Bulimia nervosa | has uncontrolled overeating followed by guilt, starving, and vomiting to lose weight. | 0.223 (0.149 0.311) |  |  | ○ |  |  |  |  |  |  |  |  |
| 98 | Symptomatic attention deficit hyperactivity disorder | Attention deficit hyperactivity disorder | is hyperactive and has difficulty concentrating, remembering things, and completing tasks. | 0.045 (0.028 0.066) |  |  | ○ |  |  |  |  |  | ○ | ○ |  |
| 99 | Symptomatic conduct disorder | Conduct disorder | has frequent behavior problems, which are sometimes violent. The person often has difficulty interacting with other people and feels irritable. | 0.241 (0.159 0.341) |  |  | ○ |  |  |  |  |  | ○ |  |  |
| 100 | Borderline intellectual disability due to other chromosomal abnormalities | Borderline intellectual functioning | is slow in learning at school. As an adult, the person has some difficulty doing complex or unfamiliar tasks but otherwise functions independently. | 0.011 (0.005 0.020) |  |  |  |  |  |  |  |  | ○ | ○ |  |
| 101 | Developmental delay or mild intellectual disability due to congenital diaphragmatic hernia | Intellectual disability/mental retardation, mild | has low intelligence, and is slow in learning at school. As an adult, the person can live independently, but often needs help to raise children and can only work at simple supervised jobs. | 0.043 (0.026 0.064) |  |  |  |  |  |  |  |  | ○ | ○ |  |
| 102 | Moderate intellectual disability due to other chromosomal abnormalities | Intellectual disability/mental retardation, moderate | has low intelligence, and is slow in learning to speak and to do even simple tasks. As an adult, the person requires a lot of support to live independently and raise children. The person can only work at the simplest supervised jobs. | 0.100 (0.066 0.142) |  |  |  |  |  |  |  |  | ○ | ○ |  |
| 103 | Severe intellectual disability due to Down syndrome | Intellectual disability/mental retardation, severe | has very low intelligence and cannot speak more than a few words, needs constant supervision and help with most daily activities, and can do only the simplest tasks. | 0.160 (0.107 0.226) |  |  |  |  |  |  |  |  | ○ | ○ |  |
| 104 | Profound intellectual disability due to other chromosomal abnormalities | Intellectual disability/mental retardation, profound | has very low intelligence, has almost no language, and does not understand even the most basic requests or instructions. The person requires constant supervision and help for all activities. | 0.200 (0.133 0.283) |  |  |  |  |  |  |  |  | ○ | ○ |  |
|  | **Hearing and vision loss** |  |  |  |  |  |  |  |  |  |  |  |  |  |  |
| 105 | Mild hearing loss due to other congenital anomalies | Hearing loss, mild | has great difficulty hearing and understanding another person talking in a noisy place (for example, on an urban street). | 0.010 (0.004 0.019) |  |  |  |  |  | ○ |  |  |  |  |  |
| 106 | Moderate hearing loss due to other congenital anomalies | Hearing loss, moderate | is unable to hear and understand another person talking in a noisy place (for example, on an urban street), and has difficulty hearing another person talking even in a quiet place or on the phone. | 0.027 (0.015 0.042) |  |  |  |  |  | ○ |  |  |  |  |  |
| 107 | Severe hearing loss due to other congenital anomalies | Hearing loss, severe | is unable to hear and understand another person talking, even in a quiet place, and unable to take part in a phone conversation. Difficulties with communicating and relating to others cause emotional impact at times (for example worry or depression). | 0.158 (0.105 0.227) |  |  | ○ |  |  | ○ |  |  | ○ |  |  |
| 108 | Profound hearing loss due to other congenital anomalies | Hearing loss, profound | is unable to hear and understand another person talking, even in a quiet place, is unable to take part in a phone conversation, and has great difficulty hearing anything in any other situation. Difficulties with communicating and relating to others often cause worry, depression, and loneliness. | 0.204 (0.134 0.288) |  |  | ○ |  |  | ○ |  |  | ○ |  |  |
| 109 | Complete hearing loss due to other congenital anomalies | Hearing loss, complete | cannot hear at all in any situation, including even the loudest sounds, and cannot communicate verbally or use a phone. Difficulties with communicating and relating to others often cause worry, depression or loneliness. | 0.215 (0.144 0.307) |  |  | ○ |  |  | ○ |  |  | ○ |  |  |
| 110 | Mild hearing loss with ringing due to age related and other hearing loss | Hearing loss, mild, with ringing | has great difficulty hearing and understanding another person talking in a noisy place (for example, on an urban street), and sometimes has annoying ringing in the ears. | 0.021 (0.012 0.036) |  |  |  |  |  | ○ |  |  |  |  |  |
| 111 | Moderate hearing loss with ringing due to other congenital anomalies | Hearing loss, moderate, with ringing | is unable to hear and understand another person talking in a noisy place (for example, on an urban street), and has difficulty hearing another person talking even in a quiet place or on the phone, and has annoying ringing in the ears for more than 5 minutes at a time, almost everyday. | 0.074 (0.049 0.107) |  |  |  |  |  | ○ |  |  |  |  |  |
| 112 | Severe hearing loss with ringing due to pneumococcal meningitis | Hearing loss, severe, with ringing | is unable to hear and understand another person talking, even in a quiet place, and unable to take part in a phone conversation, and has annoying ringing in the ears for more than 5 minutes at a time, almost everyday. Difficulties with communicating and relating to others cause emotional impact at times (for example worry or depression). | 0.261 (0.175 0.360) |  |  | ○ |  |  | ○ |  |  | ○ |  |  |
| 113 | Profound hearing loss with ringing due to other congenital anomalies | Hearing loss, profound, with ringing | is unable to hear and understand another person talking, even in a quiet place, is unable to take part in a phone conversation, has great difficulty hearing anything in any other situation, and has annoying ringing in the ears for more than 5 minutes at a time, several times a day. Difficulties with communicating and relating to others often cause worry. | 0.277 (0.182 0.387) |  |  | ○ |  |  | ○ |  |  | ○ |  |  |
| 114 | Complete hearing loss with ringing due to other congenital anomalies | Hearing loss, complete, with ringing | cannot hear at all in any situation, including even the loudest sounds, and cannot communicate verbally or use a phone, and has very annoying ringing in the ears for more than half of the day. Difficulties with communicating and relating to others often cause worry, depression or loneliness. | 0.316 (0.212 0.435) |  |  | ○ |  |  | ○ |  |  | ○ |  |  |
| 115 | Mild vision impairment due to retinopathy of prematurity | Distance vision, mild impairment | has some difficulty with distance vision, for example reading signs, but no other problems with eyesight. | 0.003 (0.001 0.007) |  |  |  |  |  | ○ |  |  |  |  |  |
| 116 | Moderate vision impairment due to other vision loss | Distance vision, moderate impairment | has vision problems that make it difficult to recognize faces or objects across a room. | 0.031 (0.019 0.049) |  |  |  |  |  | ○ |  |  |  |  |  |
| 117 | Severe vision impairment due to other vision loss | Distance vision, severe impairment | has severe vision loss, which causes difficulty in daily activities, some emotional impact (for example worry), and some difficulty going outside the home without assistance. | 0.184 (0.125 0.258) |  |  | ○ |  |  | ○ |  |  | ○ |  |  |
| 118 | Blindness due to other vision loss | Distance vision, blindness | is completely blind, which causes great difficulty in some daily activities, worry and anxiety, and great difficulty going outside the home without assistance. | 0.187 (0.124 0.260) |  |  | ○ |  |  | ○ |  |  | ○ |  |  |
| 119 | Monocular distance vision loss due to encephalitis | Distance vision, monocular | is blind in one eye and has difficulty judging distances. | 0.017 (0.009 0.029) |  |  |  |  |  | ○ |  |  |  |  |  |
| 120 | Presbyopia | Presbyopia | has difficulty seeing things that are nearer than 3 feet, but has no difficulty with seeing things at a distance. | 0.011 (0.005 0.020) |  |  |  |  |  | ○ |  |  |  |  |  |
|  | **Musculoskeletal disorders** |  |  |  |  |  |  |  |  |  |  |  |  |  |  |
| 121 | Mild low back pain without leg pain | Low back pain, mild | has mild back pain, which causes some difficulty dressing, standing, and lifting things. | 0.020 (0.011 0.035) |  | ○ |  |  |  |  |  |  | ○ |  |  |
| 122 | Moderate low back pain without leg pain | Low back pain, moderate | has moderate back pain, which causes difficulty dressing, sitting, standing, walking, and lifting things. | 0.054 (0.035 0.079) |  | ○ |  |  |  |  |  |  | ○ |  |  |
| 123 | Severe low back pain without leg pain | Low back pain, severe, without leg pain | has severe back pain, which causes difficulty dressing, sitting, standing, walking, and lifting things. The person sleeps poorly and feels worried. | 0.272 (0.182 0.373) |  | ○ | ○ |  |  |  |  |  | ○ |  | ○ |
| 124 | Severe low back pain with leg pain | Low back pain, severe, with leg pain | has severe back and leg pain, which causes difficulty dressing, sitting, standing, walking, and lifting things. The person sleeps poorly and feels worried. | 0.325 (0.219 0.446) |  | ○ | ○ |  |  |  |  |  | ○ |  | ○ |
| 125 | Most severe low back pain without leg pain | Low back pain, most severe, without leg pain | has constant back pain, which causes difficulty dressing, sitting, standing, walking, and lifting things. The person sleeps poorly, is worried, and has lost some enjoyment in life. | 0.372 (0.250 0.506) |  | ○ | ○ |  |  |  |  |  | ○ |  | ○ |
| 126 | Most severe low back pain with leg pain | Low back pain, most severe, with leg pain | has constant back and leg pain, which causes difficulty dressing, sitting, standing, walking, and lifting things. The person sleeps poorly, is worried, and has lost some enjoyment in life. | 0.384 (0.256 0.518) |  | ○ | ○ |  |  |  |  |  | ○ |  | ○ |
| 127 | Mild neck pain | Neck pain, mild | has neck pain, and has difficulty turning the head and lifting things. | 0.053 (0.034 0.078) |  | ○ |  |  |  |  |  |  |  |  |  |
| 128 | Moderate neck pain | Neck pain, moderate | has constant neck pain, and has difficulty turning the head, holding arms up, and lifting things. | 0.114 (0.075 0.162) |  | ○ |  |  |  |  |  |  | ○ |  |  |
| 129 | Severe neck pain | Neck pain, severe | has severe neck pain, and difficulty turning the head and lifting things. The person gets headaches and arm pain, sleeps poorly, and feels tired and worried. | 0.229 (0.153 0.317) |  | ○ | ○ |  |  |  |  |  | ○ |  | ○ |
| 130 | Most severe neck pain | Neck pain, most severe | has constant neck pain and arm pain, and difficulty turning the head, holding arms up, and lifting things. The person gets headaches, sleeps poorly, and feels tired and worried. | 0.304 (0.202 0.415) |  | ○ | ○ |  |  |  |  |  | ○ |  | ○ |
| 131 | Other musculoskeletal disorders severity level 1 | Musculoskeletal problems, legs, mild | has pain in the leg, which causes some difficulty running, walking long distances, and getting up and down. | 0.023 (0.013 0.037) | ○ | ○ |  |  |  |  |  |  |  |  |  |
| 132 | Moderate reduced mobility due to Guinea worm emergence | Musculoskeletal problems, legs, moderate | has moderate pain in the leg, which makes the person limp, and causes some difficulty walking, standing, lifting and carrying heavy things, getting up and down and sleeping. | 0.079 (0.054 0.110) | ○ | ○ |  |  |  |  |  |  |  |  | ○ |
| 133 | Other musculoskeletal disorders severity level 4 | Musculoskeletal problems, legs, severe | has severe pain in the leg, which makes the person limp and causes a lot of difficulty walking, standing, lifting and carrying heavy things, getting up and down, and sleeping. | 0.165 (0.112 0.232) | ○ | ○ |  |  |  |  |  |  |  |  | ○ |
| 134 | Other musculoskeletal disorders severity level 2 | Musculoskeletal problems, arms, mild | has mild pain and stiffness in the arms and hands. The person has some difficulty lifting, carrying and holding things. | 0.028 (0.017 0.045) |  | ○ |  |  |  |  |  |  | ○ |  |  |
| 135 | Other musculoskeletal disorders severity level 3 | Musculoskeletal problems, arms, moderate | has moderate pain and stiffness in the arms and hands, which causes difficulty lifting, carrying, and holding things, and trouble sleeping because of the pain. | 0.117 (0.080 0.163) |  | ○ |  |  |  |  |  |  | ○ |  | ○ |
| 136 | Other musculoskeletal disorders severity level 5 | Musculoskeletal problems, generalized, moderate | has pain and deformity in most joints, causing difficulty moving around, getting up and down, and using the hands for lifting and carrying. The person often feels fatigue. | 0.317 (0.216 0.440) | ○ | ○ |  | ○ |  |  |  |  |  |  |  |
| 137 | Other musculoskeletal disorders severity level 6 | Musculoskeletal problems, generalized, severe | has severe, constant pain and deformity in most joints, causing difficulty moving around, getting up and down, eating, dressing, lifting, carrying and using the hands. The person often feels sadness, anxiety and extreme fatigue. | 0.581 (0.403 0.739) | ○ | ○ | ○ |  | ○ |  |  |  | ○ |  |  |
| 138 | Symptomatic episodes of gout | Gout, acute | has severe pain and swelling in the leg, making it very difficult to get up and down, stand, walk, lift, and carry heavy things. The person has trouble sleeping because of the pain. | 0.295 (0.196 0.409) | ○ | ○ |  |  |  |  |  |  |  |  | ○ |
|  | **Injury** |  |  |  |  |  |  |  |  |  |  |  |  |  |  |
| 139 | Injuries | Amputation of one upper limb (long term, without treatment) | has lost one hand and part of the arm, leaving pain and tingling in the stump. The person needs help from others to lift objects or do daily activities such as cooking. | 0.105 (0.085 0.128) |  | ○ |  |  | ○ |  |  |  | ○ |  |  |
| 140 | Injuries | Concussion (short term) | has episodes of headaches, dizziness, nausea and difficulty concentration. | 0.104 (0.085 0.126) |  | ○ |  |  |  |  |  |  |  | ○ | ○ |
| 141 | Guillain Barre syndrome due to other neurological disorders | Spinal cord lesion, below neck level (treated) | is paralyzed from the waist down, cannot feel or move the legs and has difficulties with urine and bowel control. The person uses a wheelchair to move around. | 0.296 (0.198 0.414) | ○ |  |  |  |  |  |  |  |  |  | ○ |
|  | **Other** |  |  |  |  |  |  |  |  |  |  |  |  |  |  |
| 142 | Menstrual disorders without anemia | Abdominopelvic problem, mild | has some pain in the belly that causes nausea but does not interfere with daily activities. | 0.011 (0.005 0.021) |  | ○ |  |  |  |  |  |  |  |  | ○ |
| 143 | Moderate other gynecological disorders | Abdominopelvic problem, moderate | has pain in the belly and feels nauseous. The person has difficulties with daily activities. | 0.114 (0.078 0.159) |  | ○ |  |  |  |  |  |  | ○ |  | ○ |
| 144 | Severe other gynecological disorders | Abdominopelvic problem, severe | has severe pain in the belly and feels nauseous. The person is anxious and unable to carry out daily activities. | 0.324 (0.220 0.442) |  |  |  | ○ |  |  |  |  |  |  |  |
| 145 | Mild anemia due to endocrine, metabolic, blood, and immune disorders | Anemia, mild | feels slightly tired and weak at times, but this does not interfere with normal daily activities. | 0.004 (0.001 0.008) |  |  |  | ○ |  |  |  |  | ○ |  |  |
| 146 | Moderate anemia due to endocrine, metabolic, blood, and immune disorders | Anemia, moderate | feels moderate fatigue, weakness, and shortness of breath after exercise, making daily activities more difficult. | 0.052 (0.034 0.076) |  |  |  | ○ |  |  |  |  | ○ |  |  |
| 147 | Severe anemia due to endocrine, metabolic, blood, and immune disorders | Anemia, severe | feels very weak, tired and short of breath, and has problems with activities that require physical effort or deep concentration. | 0.149 (0.101 0.209) |  |  |  |  |  |  |  |  |  |  |  |
| 148 | Chronic periodontal diseases | Periodontitis | has minor bleeding of the gums from time to time, with mild discomfort. | 0.007 (0.003 0.014) |  | ○ |  |  |  |  |  |  |  |  | ○ |
| 149 | Pain due to caries of permanent teeth | Dental caries: symptomatic | has a toothache, which causes some difficulty in eating. | 0.010 (0.005 0.019) |  | ○ |  |  |  |  |  |  |  |  |  |
| 150 | Difficulty eating due to edentulism and severe tooth loss | Severe tooth loss | has lost more than 20 teeth including front and back, and has great difficulty in eating meat, fruits, and vegetables. | 0.067 (0.045 0.095) |  |  |  |  |  |  |  |  | ○ |  |  |
| 151 | Hirsutism due to polycystic ovarian syndrome | Disfigurement, level 1 | has a slight, visible physical deformity that others notice, which causes some worry and discomfort. | 0.011 (0.005 0.021) |  |  |  |  | ○ |  |  |  |  |  |  |
| 152 | Disfigurement level 2 due to congenital limb deficiency | Disfigurement, level 2 | has a visible physical deformity that causes others to stare and comment. As a result, the person is worried and has trouble sleeping and concentrating. | 0.067 (0.044 0.096) |  |  | ○ |  | ○ |  |  |  |  |  | ○ |
| 153 | Severe skin disease without itch due to onchocerciasis | Disfigurement, level 3 | has an obvious physical deformity that makes others uncomfortable, which causes the person to avoid social contact, feel worried, sleep poorly, and think about suicide. | 0.405 (0.275 0.546) |  |  | ○ |  | ○ |  |  |  |  |  | ○ |
| 154 | Mild decubitus ulcer | Disfigurement, level 1, with itch or pain | has a slight, visible physical deformity that is sometimes sore or itchy. Others notice the deformity, which causes some worry and discomfort. | 0.027 (0.015 0.042) |  | ○ | ○ |  | ○ |  |  |  |  |  |  |
| 155 | Disfigurement level 2 with pain due to congenital limb deficiency | Disfigurement, level 2, with itch or pain | has a visible physical deformity that is sore and itchy. Other people stare and comment, which causes the person to worry. The person has trouble sleeping and concentrating. | 0.188 (0.125 0.267) |  | ○ | ○ |  | ○ |  |  |  |  |  | ○ |
| 156 | Severe psoriasis | Disfigurement, level 3, with itch or pain | has an obvious physical deformity that is very painful and itchy. The physical deformity makes others uncomfortable, which causes the person to avoid social contact, feel worried, sleep poorly, and think about suicide. | 0.576 (0.401 0.731) |  | ○ | ○ |  | ○ |  |  |  |  |  | ○ |
| 157 | Treated heart failure due to endocrine, metabolic, blood, and immune disorders | Generic uncomplicated disease: worry and daily medication | has a chronic disease that requires medication every day and causes some worry but minimal interference with daily activities. | 0.049 (0.031 0.072) |  |  | ○ |  |  |  |  |  |  |  |  |
| 158 | Hemoglobin SC disease, without anemia | Generic uncomplicated disease: anxiety about diagnosis | has a disease diagnosis that causes some worry but minimal interference with daily activities. | 0.012 (0.006 0.023) |  |  | ○ |  |  |  |  |  |  |  |  |
| 159 | Moderate wasting with edema | Kwashiorkor | is very tired and irritable and has diarrhea. | 0.051 (0.031 0.079) |  |  | ○ | ○ |  |  | ○ |  |  |  |  |
| 160 | Severe wasting without edema | Severe wasting | is extremely skinny and has no energy. | 0.128 (0.082 0.183) |  |  |  | ○ |  |  |  |  |  |  | ○ |
| 161 | Speech problems due to motor neuron disease | Speech problems | has difficulty speaking, and others find it difficult to understand. | 0.051 (0.032 0.078) |  |  |  |  |  |  |  |  |  |  | ○ |
| 162 | Mild motor impairment due to spina bifida | Motor impairment, mild | has some difficulty in moving around but is able to walk without help. | 0.010 (0.005 0.019) | ○ |  |  |  |  |  |  |  |  |  |  |
| 163 | Moderate motor impairment due to hemolytic disease and other neonatal jaundice | Motor impairment, moderate | has some difficulty in moving around, and difficulty in lifting and holding objects, dressing and sitting upright, but is able to walk without help. | 0.061 (0.040 0.089) | ○ |  |  |  |  |  |  |  | ○ |  |  |
| 164 | Severe motor impairment due to spina bifida | Motor impairment, severe | is unable to move around without help, and is not able to lift or hold objects, get dressed or sit upright. | 0.402 (0.268 0.545) | ○ |  |  |  |  |  |  |  | ○ |  |  |
| 165 | Mild motor impairment and mild intellectual disability due to encephalocele | Motor plus cognitive impairments, mild | has some difficulty in moving around but is able to walk without help. The person is slow in learning at school. As an adult, the person has some difficulty doing complex or unfamiliar tasks but otherwise functions independently. | 0.031 (0.018 0.050) | ○ |  |  |  |  |  |  |  |  | ○ |  |
| 166 | Moderate motor impairment and moderate intellectual disability due to spina bifida | Motor plus cognitive impairments, moderate | has some difficulty in moving around, holding objects, dressing and sitting upright, but can walk without help. The person has low intelligence and is slow in learning to speak and to do simple tasks. | 0.203 (0.134 0.290) | ○ |  |  |  |  |  |  |  | ○ | ○ |  |
| 167 | Severe motor and cognitive impairment due to Edward Syndrome or Patau Syndrome | Motor plus cognitive impairments, severe | cannot move around without help, and cannot lift or hold objects, get dressed or sit upright. The person also has very low intelligence, speaks few words, and needs constant supervision and help with all daily activities. | 0.542 (0.374 0.702) | ○ |  |  |  |  |  |  |  | ○ | ○ |  |
| 168 | Rectovaginal fistula | Rectovaginal fistula | has an abnormal opening between her vagina and rectum causing flatulence and feces to escape through the vagina. The person gets infections in her vagina, and has pain when urinating. | 0.501 (0.339 0.657) |  | ○ |  |  |  |  | ○ |  |  |  | ○ |
| 169 | Vesicovaginal fistula | Vesicovaginal fistula | has an abnormal opening between the bladder and the vagina, which makes her unable to control urinating. The woman is anxious and depressed. | 0.342 (0.227 0.478) |  |  | ○ |  |  |  |  |  |  |  |  |
| 170 | Severe endocrine, metabolic, blood, and immune disorders | Thrombocytopenic purpura | easily bruises and sometimes bleeds from the gums and nose; feels weak and has some difficulty with daily activities. | 0.159 (0.106 0.226) |  |  |  | ○ |  |  |  |  | ○ |  | ○ |
| 171 | Mild endocrine, metabolic, blood, and immune disorders | Hypothyroidism | has low energy and feels cold. | 0.019 (0.010 0.032) |  |  |  | ○ |  |  |  |  |  |  | ○ |
| 172 | Moderate endocrine, metabolic, blood, and immune disorders | Hyperthyroidism | feels nervous, has palpitations, sweats a lot and has difficulty sleeping. | 0.145 (0.096 0.202) |  |  | ○ |  |  |  |  |  |  |  | ○ |
| 173 | Musculoskeletal disorders | Neck pain, moderate | has moderately severe neck pain, and difficulty turning the head and lifting things. The person gets headaches. | 0.056 (0.044 0.067) |  | ○ |  |  |  |  |  |  | ○ |  |  |
| 174 | Musculoskeletal disorders | Osteomyelitis | has severe pain in one leg causing difficulty in moving about. | 0.053 (0.041 0.065) | ○ | ○ |  |  |  |  |  |  |  |  |  |
| 175 | Musculoskeletal disorders | Shoulder lesions | has a painful shoulder that causes difficulty in using the arm. | 0.016 (0.012 0.02) | ○ |  |  |  |  |  |  |  |  |  |  |
| 176 | Diabetes, digestive, and genitourinary disease | Heart burn & reflux “GERD” | A couple of times a day, this person has a burning sensation in the back of the chest as contents from the stomach moves back into the oesophagus. | 0.038 (0.029 0.046) |  | ○ |  |  |  |  |  |  |  |  |  |
| 177 | Diabetes, digestive, and genitourinary disease | Constipation | this person passes stools infrequently and when it does happen it is painful | 0.075 (0.061 0.092) |  | ○ |  |  |  |  |  |  |  |  | ○ |
| 178 | Diabetes, digestive, and genitourinary disease | vaginal discharge | (women) has a vaginal discharge that sometimes causes itch. | 0.018 (0.013 0.022) |  | ○ |  |  |  |  |  |  |  |  | ○ |
| 179 | Diabetes, digestive, and genitourinary disease | dyspareunia | (women) experiences pain during sexual intercourse. | 0.022 (0.017 0.027) |  | ○ |  |  |  |  |  |  |  |  |  |
| 180 | Diabetes, digestive, and genitourinary disease | Stress incontinence | loses small amounts of urine without meaning to when coughing, sneezing, laughing or during physical exercise. | 0.032 (0.024 0.038) |  |  |  |  |  |  |  |  |  |  | ○ |
| 181 | Diabetes, digestive, and genitourinary disease | Irritable bowel syndrome | experiences abdominal pain, bloating, stomach rumbling, flatulence and an irregular bowel pattern. | 0.062 (0.05 0.077) |  | ○ |  |  |  |  |  |  |  |  | ○ |
| 182 | Mental, behavioural, and substance abuse disorder | somatoform disorder | suffers from multiple symptoms of pain, stomach complaints or nerve problems for which no explanation can be found. The symptoms are real to the person and a source of worry. | 0.144 (0.116 0.174) |  | ○ | ○ |  |  |  |  |  |  |  |  |
| 183 | Mental, behavioural, and substance abuse disorder | borderline personality disorder | has unstable moods, often does things without thinking about the consequences and has a tendency to view others and self as either very good or very bad. This leads to chaotic interpersonal relationships and causes the person to feel angry, anxious or depressed. | 0.193 (0.16 0.228) |  |  | ○ |  |  |  |  |  |  |  |  |
| 184 | Mental, behavioural, and substance abuse disorder | harmful alcohol use | regularly gets drunk putting the person at risk of injuries and other harm to health. | 0.106 (0.087 0.132) |  |  |  |  |  |  |  | ○ |  |  |  |
| 185 | Neurological disorders | vertigo and balance disorder (Menière, labyrinthitis) | has short spells of dizziness and losing balance; when these spells are not there the person is worried it may happen again | 0.097 (0.079 0.119) |  |  | ○ |  |  |  |  |  |  |  | ○ |
| 186 | Neurological disorders | trigeminal neuralgia | has episodes of severe pain in the face | 0.068 (0.056 0.084) |  | ○ |  |  |  |  |  |  |  |  |  |
| 187 | Neurological disorders | Encephalopathy - moderate | has difficulty concentrating, confusion, fatigue and irritability. The person has difficulty with walking, speaking and personal care. | 0.410 (0.358 0.47) | ○ |  | ○ | ○ |  |  |  |  |  |  |  |
| 188 | Neurological disorders | Encephalopathy - severe | has difficulty concentrating or speaking, confusion, fatigue and irritability. The person needs a great deal of help from others to do even basic daily activities such as eating and using the toilet, and the person is very limited in other activities. | 0.447 (0.391 0.501) |  |  | ○ | ○ |  |  |  |  | ○ |  |  |
| 189 | Infectious diseases | Thrombocytopenic purpura | has bruising on the extremities and bleeding of the gums and nose and feels very weak. | 0.167 (0.134 0.201) |  |  |  | ○ |  |  |  |  | ○ |  | ○ |
| 190 | Infectious diseases | Lymphogranuloma Venereum - local infection | has a painful lump in the groin. | 0.070 (0.057 0.087) |  | ○ |  |  |  |  |  |  |  |  |  |
| 191 | Infectious diseases | Subacute sclerosing panencephalitis - phase 1 | has difficulty concentrating, some memory problems, confusion and irritability. | 0.088 (0.07 0.108) |  |  | ○ |  |  |  |  |  |  | ○ |  |
| 192 | Infectious diseases | Subacute sclerosing panencephalitis - phase 2 | has difficulty concentrating, some memory problems, confusion and irritability. The person has difficulty controlling body movements and moving around. | 0.276 (0.235 0.323) | ○ |  | ○ |  |  |  |  |  |  | ○ |  |
| 193 | Infectious diseases | Subacute sclerosing panencephalitis - phase 3 | is often asleep or unconscious; when awake cannot think or see clearly. The person needs a great deal of help from others to do even basic daily activities such as eating and using the toilet, and the person is very limited in other activities. | 0.543 (0.481 0.606) |  |  |  |  |  | ○ |  |  | ○ |  | ○ |
| 194 | Other | Haemorrhoids | loses some blood when passing stools and at times has pain around the anus. | 0.109 (0.085 0.133) |  | ○ |  |  |  |  |  |  |  |  | ○ |
| 195 | Other | anal fissure/abcess/fistula | has pain around the anus that is worse when passing stools and sitting. | 0.082 (0.066 0.1) |  | ○ |  |  |  |  |  |  |  |  |  |
| 196 | Other | Hyperthyroidism | is feels nervous, has palpitations, sweats a lot and has difficulty sleeping. | 0.144 (0.115 0.176) |  |  | ○ |  |  |  |  |  |  |  | ○ |
| 197 | Other | Allergic rhinitis (hay fever) | has an itchy running nose and frequently sneezes. | 0.006 (0.004 0.009) |  |  |  |  |  |  |  |  |  |  | ○ |
| 198 | Other | Varicose veins | has swollen veins in the legs that sometimes cause some swelling of the feet. | 0.020 (0.016 0.025) |  | ○ |  |  |  |  |  |  |  |  |  |
| 199 | Other | Carpal tunnel syndrome | feels pain, numbness or weakness in part of the hand during some activities such as knitting, driving or typing. | 0.039 (0.031 0.047) |  | ○ |  |  |  |  |  |  | ○ |  |  |
| 200 | Other | Intensive care unit admission | is very ill, confined to bed; often unconscious or asleep, unable to speak and completely dependent on others. | 0.655 (0.579 0.727) |  |  | ○ |  |  |  |  |  | ○ |  | ○ |
| 201 | Other | Invasive device/drain | carries a drain, which may irritate locally and also hinders the patient when moving around. | 0.163 (0.131 0.198) | ○ | ○ |  |  |  |  |  |  |  |  |  |
| 202 | Other | insomnia | has difficulty falling or staying asleep. | 0.023 (0.017 0.028) |  |  |  |  |  |  |  |  |  |  | ○ |
| 203 | Other | sleep apnoea | while sleeping has short episodes of abnormally low breathing; the person does not notice this but may feel tired or sleepy during the day. | 0.036 (0.027 0.044) |  |  |  |  |  |  |  |  |  |  | ○ |
| 204 | Other | Hypothyroidism | feels tired, has gained weight and feels cold. | 0.022 (0.017 0.028) |  |  |  | ○ |  |  |  |  |  |  | ○ |
| 205 | Infectious diseases, moderate hearing loss due to other bacterial meningitis | Hearing loss, moderate | is unable to hear and understand others talking in a noisy place (for example, on an urban street), even in a quiet place or on the phone. | 0.027 (0.015 0.042) |  |  |  |  |  | ○ |  |  |  |  |  |
| 206 | Infectious diseases, severe hearing loss due to meningococcal meningitis | Hearing loss, severe | is unable to hear and understand others talking, even in a quiet place, and unable to talk on the phone. Difficulties in communicating with others and socializing cause emotional impact at times (for example anxiety or depression). | 0.158 (0.105 0.227) |  |  | ○ |  |  | ○ |  |  | ○ |  |  |

GBD: Global Burden of Disease study; DW: Disability Weight; ADL: activities of daily living; Others: other physical symptoms, including dyspnoea, nausea, palpitations, reduced appetite, sleeping.

**Appendix table 3: 28 health states used in population health equivalence method**^1^

| id | Health state |
| --- | --- |
|  |  |
| 1 | Asthma, controlled |
| 2 | Hearing loss, moderate |
| 3 | Asthma, partially controlled |
| 4 | Chronic obstructive pulmonary disease (COPD) and other chronic respiratory problems, mild |
| 5 | Distance vision, moderate impairment |
| 6 | Musculoskeletal problems, legs, mild |
| 7 | Angina pectoris, moderate |
| 8 | Hearing loss, complete |
| 9 | Diabetic neuropathy |
| 10 | Dementia, mild |
| 11 | Anemia, moderate |
| 12 | Anxiety disorders, moderate |
| 13 | Amputation of finger(s), excluding thumb: long term, with treatment |
| 14 | Acute myocardial infarction, days 3-28 |
| 15 | Stroke, long term consequences, moderate |
| 16 | Traumatic brain injury: long-term consequences, moderate, with or without treatment |
| 17 | Motor impairment, moderate |
| 18 | Cancer, diagnosis and primary therapy |
| 19 | Major depressive disorder, moderate episode |
| 20 | Decompensated cirrhosis of the liver |
| 21 | Distance vision, severe impairment |
| 22 | Parkinson disease, moderate |
| 23 | Anxiety disorders, severe |
| 24 | COPD and other chronic respiratory problems, severe |
| 25 | Dementia, moderate |
| 26 | Multiple sclerosis, severe |
| 27 | Stroke, long term consequences, severe plus cognition problems |
| 28 | Spinal cord lesion at neck level: treated |


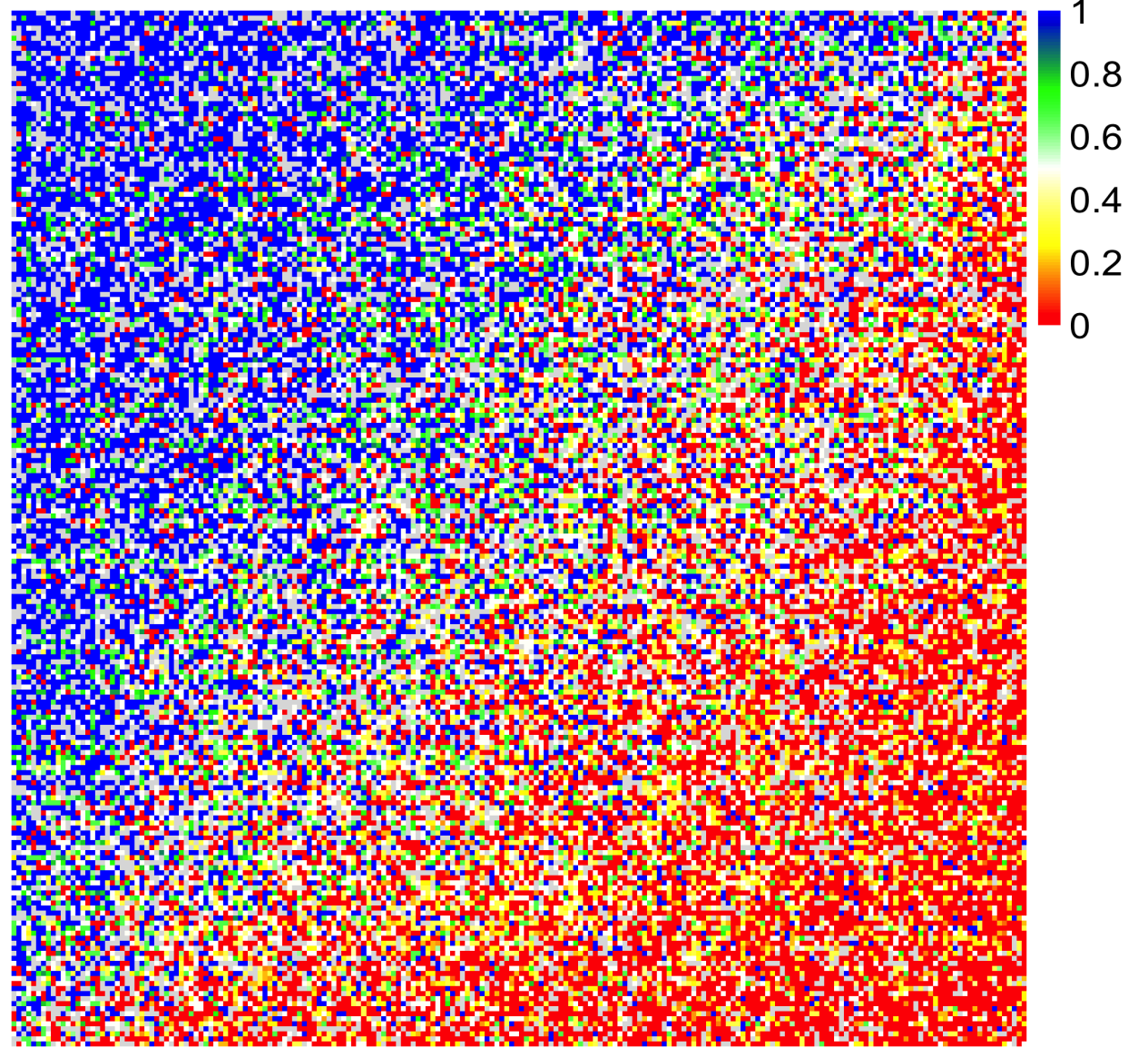


**Appendix figure 1: Response probabilities for paired comparisons in Wuhan population.** Colors on the heat maps correspond to the probability that the first health state in a paired comparison was chosen as the healthier outcome. Variation in the amount of measurement error across surveys is reflected in the varying degrees to which response probabilities follow an orderly transition from high to low between the upper left and the lower right corners in each heat map. A heat map with no measurement error and high internal consistency indicated by a smooth color transition from blue to red along the diagonal, whereas a heat map with a high amount of error would have a completely random assortment of colored squares.

**References**

1. Liu X, Wang F, Zhou M, Yu Y, Qi J, Yin P, Yu S, Zhou Y, Lin L, Liu Y, Wang Q, Zhong W, Huang S, Li Y, Liu L, Liu Y, Ma F, Zhang Y, Tian Y, Yu Q, Zeng J, Pan J, Zhou M, Kang W, Zhou JY, Yu H, Liu Y, Li S, Yu H, Wang C, Xia T, Xi J, Ren X, Xing X, Cheng Q, Fei F, Wang D, Zhang S, He Y, Wen H, Liu Y, Shi F, Wang Y, Sun P, Bai J, Wang X, Shen H, Ma Y, Yang D, Mubarik S, Cao J, Meng R, Zhang Y, Guo Y, Yan Y, Zhang W, Ke S, Zhang R, Wang D, Zhang T, Nomura S, Hay SI, Salomon JA, Haagsma JA, Murray CJL, Vos T, Yu C. Eliciting national and subnational sets of disability weights in mainland China: Findings from the Chinese disability weight measurement study. *The Lancet Regional Health - Western Pacific* 2022;26:100520. <https://doi.org/10.1016/j.lanwpc.2022.100520>

2. Salomon JA, Haagsma JA, Davis A, et al. Disability weights for the Global Burden of Disease 2013 study. *Lancet Global Health* 2015; 3(11): e712-23.
